# Decoding the historical tale: COVID-19 impact on haematological malignancy patients—EPICOVIDEHA insights from 2020 to 2022

**DOI:** 10.1016/j.eclinm.2024.102553

**Published:** 2024-03-18

**Authors:** Jon Salmanton-García, Francesco Marchesi, Francesca Farina, Barbora Weinbergerová, Federico Itri, Julio Dávila-Valls, Sonia Martín-Pérez, Andreas Glenthøj, Ditte Stampe Hersby, Maria Gomes da Silva, Raquel Nunes Rodrigues, Alberto López-García, Raúl Córdoba, Yavuz M. Bilgin, Iker Falces-Romero, Shaimaa El-Ashwah, Ziad Emarah, Caroline Besson, Milena Kohn, Jaap Van Doesum, Emanuele Ammatuna, Monia Marchetti, Jorge Labrador, Giovanni Paolo Maria Zambrotta, Luisa Verga, Ozren Jaksic, Marcio Nucci, Klára Piukovics, Alba Cabirta-Touzón, Moraima Jiménez, Elena Arellano, Ildefonso Espigado, Ola Blennow, Anna Nordlander, Stef Meers, Jens van Praet, Tommaso Francesco Aiello, Carolina Garcia-Vidal, Nicola Fracchiolla, Mariarita Sciumè, Guldane Cengiz Seval, Pavel Žák, Caterina Buquicchio, Carlo Tascini, Stefanie K. Gräfe, Martin Schönlein, Tatjana Adžić-Vukičević, Valentina Bonuomo, Chiara Cattaneo, Summiya Nizamuddin, Martin Čerňan, Gaëtan Plantefeve, Romane Prin, Tomas Szotkovski, Graham P. Collins, Michelina Dargenio, Verena Petzer, Dominik Wolf, Natasha Čolović, Lucia Prezioso, Toni Valković, Francesco Passamonti, Gustavo-Adolfo Méndez, Uluhan Sili, Antonio Vena, Martina Bavastro, Alessandro Limongelli, Rafael F. Duarte, Marie-Pierre Ledoux, Milche Cvetanoski, Zlate Stojanoski, Marina Machado, Josip Batinić, Gabriele Magliano, Monika M. Biernat, Nikola Pantić, Christian Bjørn Poulsen, Annarosa Cuccaro, Maria Ilaria Del Principe, Austin Kulasekararaj, Irati Ormazabal-Vélez, Alessandro Busca, Fatih Demirkan, Marriyam Ijaz, Nikolai Klimko, Igor Stoma, Sofya Khostelidi, Noemí Fernández, Ali S. Omrani, Rui Bergantim, Nick De Jonge, Guillemette Fouquet, Milan Navrátil, Ghaith Abu-Zeinah, Michail Samarkos, Johan Maertens, Cristina De Ramón, Anna Guidetti, Ferenc Magyari, Tomás José González-López, Tobias Lahmer, Olimpia Finizio, Natasha Ali, László Imre Pinczés, Esperanza Lavilla-Rubira, Alessandra Romano, Maria Merelli, Mario Delia, Maria Calbacho, Joseph Meletiadis, Darko Antić, José-Ángel Hernández-Rivas, Joyce Marques de Almeida, Murtadha Al-Khabori, Martin Hoenigl, Maria Chiara Tisi, Nina Khanna, Aleksandra Barać, Noha Eisa, Roberta Di Blasi, Raphaël Liévin, Carolina Miranda-Castillo, Nathan C. Bahr, Sylvain Lamure, Mario Virgilio Papa, Ayel Yahya, Avinash Aujayeb, Jan Novák, Nurettin Erben, María Fernández-Galán, José-María Ribera-Santa Susana, Ikhwan Rinaldi, Rita Fazzi, Monica Piedimonte, Rémy Duléry, Yung Gonzaga, Andrés Soto-Silva, Giuseppe Sapienza, Alexandra Serris, Ľuboš Drgoňa, Ana Groh, Laura Serrano, Eleni Gavriilaki, Athanasios Tragiannidis, Juergen Prattes, Nicola Coppola, Vladimir Otašević, Miloš Mladenović, Mirjana Mitrović, Bojana Mišković, Pavel Jindra, Sofia Zompi, Maria Vittoria Sacchi, Carolin Krekeler, Maria Stefania Infante, Daniel García-Bordallo, Gökçe Melis Çolak, Jiří Mayer, Marietta Nygaard, Michaela Hanáková, Zdeněk Ráčil, Matteo Bonanni, Philipp Koehler, Laman Rahimli, Oliver A. Cornely, Livio Pagano, EPICOVIDEHA registry, Francisco Javier Martín-Vallejo, Przemyslaw Zdziarski, Hossein Zarrinfer, Jana Wittig, Sein Win, Vivien Wai-Man, Benjamín Víšek, Donald C. Vinh, Maria Vehreschild, Gina Varricchio, Panagiotis Tsirigotis, Ana Torres-Tienza, Alina Daniela Tanase, Agostino Tafuri, Maria Stamouli, Jiří Sramek, Carole Soussain, Ayten Shirinova, Jörg Schubert, Enrico Schalk, Mohammad Reza Salehi, Modar Saleh, Giorgio Rosati, Elisa Roldán, Florian Reizine, Mayara Rêgo, Isabel Regalado-Artamendi, Marina Popova, Fernando Pinto, Laure Philippe, Hans Martin Orth, Hans-Beier Ommen, Aleš Obr, Lucía Núñez-Martín-Buitrago, Nicolas Noël, Julia Neuhann, Gianpaolo Nadali, Julia A. Nacov, Ana M. Munhoz Alburquerque, Maria Enza Mitra, Malgorzata Mikulska, Sibylle Mellinghoff, Ben Mechtel, Juan-Alberto Martín-González, Sandra Malak, Jorge Loureiro-Amigo, Lisset Lorenzo De La Peña, Giulia Liberti, Marianne Landau, Ira Lacej, Martin Kolditz, Chi Shan Kho, Reham Abdelaziz Khedr, Meinolf Karthaus, Linda Katharina Karlsson, María-Josefa Jiménez-Lorenzo, Macarena Izuzquiza, Baerbel Hoell-Neugebauer, Raoul Herbrecht, Christopher H. Heath, Fabio Guolo, Jan Grothe, Antonio Giordano, Sergey Gerasymchuk, Ramón García-Sanz, Nicole García-Poutón, Vaneuza Araújo Moreira Funke, Monica Fung, Charlotte Flasshove, Luana Fianchi, Jenna Essame, Matthias Egger, Bernard Drenou, Giulia Dragonetti, Maximilian Desole, Roberta Della Pepa, Bénédicte Deau Fischer, Elizabeth De Kort, Erik De Cabo, François Danion, Etienne Daguindau, Tania Cushion, Louise Cremer, Marianna Criscuolo, Gregorio Cordini, Antonella Cingolani, Fabio Ciceri, Fazle Rabbi Chowdhury, Ekaterina Chelysheva, Adrien Chauchet, Louis Yi Ann Chai, M. Mansour Ceesay, Elena Busch, Mathias Brehon, Davimar M.M. Borducchi, Stephen Booth, Serge Bologna, Caroline Berg Venemyr, Rebeca Bailén-Almorox, Anastasia Antoniadou, Amalia N. Anastasopoulou, Fevzi Altuntaş

**Affiliations:** a University of Cologne, Faculty of Medicine, and University Hospital Cologne, Institute of Translational Research, Cologne Excellence Cluster on Cellular Stress Responses in Aging-Associated Diseases (CECAD), Cologne, Germany; b University of Cologne, Faculty of Medicine, University Hospital Cologne, Department I of Internal Medicine, Center for Integrated Oncology Aachen Bonn Cologne Duesseldorf (CIO ABCD) and Excellence Center for Medical Mycology (ECMM), Cologne, Germany; c German Centre for Infection Research (DZIF), Partner Site Bonn-Cologne, Cologne, Germany; d Hematology and Stem Cell Transplant Unit, IRCCS Regina Elena National Cancer Institute, Rome, Italy; e IRCCS Ospedale San Raffaele, Milan, Italy; f Masaryk University and University Hospital Brno - Department of Internal Medicine, Hematology and Oncology, Brno, Czech Republic; g San Luigi Gonzaga Hospital - Orbassano, Orbassano, Italy; h Hospital Nuestra Señora de Sonsoles, Ávila, Spain; i Department of Hematology, Copenhagen University Hospital - Rigshospitalet, Copenhagen, Denmark; j Portuguese Institute of Oncology, Lisbon, Portugal; k Fundación Jimenez Diaz University Hospital, Health Research Institute IIS-FJD, Madrid, Spain; l Department of Internal Medicine, ADRZ, Goes, Netherlands; m Microbiology and Parasitology Department, University Hospital La Paz, Madrid, Spain; n CIBERINFEC, Instituto de Salud Carlos III, Madrid, Spain; o Oncology Center, Mansoura University, Mansoura, Egypt; p King Abdullah Medical City, Makkah, Saudi Arabia; q Centre Hospitalier de Versailles, Le Chesnay, France; r University Medical Center Groningen, Groningen, Netherlands; s Hematology and Transplant Unit, Azienda Ospedaliera SS Antonio e Biagio e Cesare Arrigo, Alessandria, Italy; t Department of Hematology, Research Unit, Hospital Universitario de Burgos, Burgos, Spain; u Azienda Ospedaliera San Gerardo - Monza, Monza, Italy; v Università Milano-Bicocca, Milan, Italy; w University Hospital Dubrava, Zagreb, Croatia; x Department of Internal Medicine, Federal University of Rio de Janeiro and Grupo Oncoclinicas, Rio de Janeiro, Brazil; y Department of Internal Medicine, South Division Faculty of Medicine University of Szeged, Szeged, Hungary; z Department of Hematology, Vall d’Hebron Hospital Universitari, Experimental Hematology, Vall d’Hebron Institute of Oncology (VHIO), Vall d’Hebron Barcelona Hospital Campus, Barcelona, Spain; aa Department of Hematology, University Hospital Virgen Macarena - University Hospital Virgen del Rocío, Instituto de Biomedicina de Sevilla (IBIS/CSIC), Universidad de Sevilla (Departamento de Medicina), Seville, Spain; ab Department of Infectious Diseases, Karolinska University Hospital, Stockholm, Sweden; ac AZ KLINA, Brasschaat, Belgium; ad Department of Nephrology and Infectious diseases, AZ Sint-Jan Brugge-Oostende AV, Brugge, Belgium; ae Department of Infectious Diseases, Hospital Clinic de Barcelona, University of Barcelona, IDIBAPS, Barcelona, Spain; af Hematology Unit, Fondazione IRCCS Ca' Granda Ospedale Maggiore Policlinico, Milan, Italy; ag Ankara University, Ankara, Turkey; ah University Hospital Hradec Králové, Hradec Králové, Czech Republic; ai Ematologia con Trapianto, Ospedale Dimiccoli Barletta, Barletta, Italy; aj Azienda Sanitaria Universitaria del Friuli Centrale, Udine, Italy; ak University Medical Center Hamburg-Eppendorf, Hamburg, Germany; al Department of Oncology, Hematology and Bone Marrow Transplantation with Section of Pneumology, University Medical Center Hamburg-Eppendorf, Hamburg, Germany; am COVID Hospital “Batajnica”, Belgrade, Serbia; an Policlinico Borgo Roma Verona, Verona, Italy; ao Hematology Unit, ASST-Spedali Civili, Brescia, Italy; ap Shaukat Khanum Memorial Cancer Hospital and Research Centre, Lahore, Pakistan; aq University Hospital Olomouc, Olomouc, Czech Republic; ar Head ICU and CRC, Centre Hospitalier Victor DUPOUY, Argenteuil, France; as CRA from CRC Centre Hospitalier Victor DUPOUY, Argenteuil, France; at Oxford University Hospitals, Oxford, United Kingdom; au Ospedale Vito Fazzi, Lecce, Italy; av Department of Hematology and Oncology, Comprehensive Cancer Center Innsbruck (CCCI), Medical University of Innsbruck (MUI), Innsbruck, Austria; aw University Clinical Center Serbia, Belgrade, Serbia; ax Hospital University of Parma - Hematology and Bone Marrow Unit, Parma, Italy; ay University Hospital Centre Rijeka, Rijeka, Croatia; az Croatian Cooperative Group for Hematological Diseases (CROHEM), Croatia; ba Faculty of Medicine and Faculty of Health Studies of University of Rijeka, Rijeka, Croatia; bb Department of Medicine and Surgery, University of Insubria and ASST Sette Laghi, Ospedale di Circolo of Varese, Varese, Italy; bc Hospital Escuela de Agudos Dr. Ramón Madariaga, Posadas, Argentina; bd Department of Infectious Diseases and Clinical Microbiology, School of Medicine, Marmara University, Istanbul, Turkey; be Ospedale Policlinico San Martino, Genoa, Italy; bf Hospital Universitario Puerta de Hierro, Majadahonda, Spain; bg ICANS, Strasbourg, France; bh University Clinic of Hematology, Skopje, North Macedonia; bi Clinical Microbiology and Infectious Diseases Department, Hospital General Universitario Gregorio Marañón, Madrid, Spain; bj University Hospital Centre Zagreb, Zagreb, Croatia; bk Faculty of Medicine University of Zagreb, Zagreb, Croatia; bl ASST Grande Ospedale Metropolitano Niguarda, Milan, Italy; bm Wroclaw Medical University, Wroclaw, Poland; bn University Clinical Center of Serbia, Belgrade, Serbia; bo Zealand University Hospital, Roskilde, Denmark; bp Hematology Unit, Center for Translational Medicine, Azienda USL Toscana NordOvest, Livorno, Italy; bq National Cancer Institute, Fondazione ‘G. Pascale’, IRCCS, Hematology-Oncology and Stem Cell Transplantation Unit, Naples, Italy; br Hematology, Department of Biomedicine and Prevention, University of Rome Tor Vergata, Rome, Italy; bs Department of Hematological Medicine, King's College Hospital NHS Foundation Trust, London, United Kingdom; bt Hospital Universitario de Navarra, Iruña-Pamplona, Spain; bu Stem Cell Transplant Center, AOU Citta’ della Salute e della Scienza, Turin, Italy; bv Dokuz Eylul University, Division of Hematology, Izmir, Turkey; bw North-Western State Medical University Named after Iliá Ilich Méchnikov, Saint-Petersburg, Russia; bx Gomel State Medical University, Gomel, Belarus; by Hospital Universitario Marqués de Valdecilla, Santander, Spain; bz Hamad Medical Corporation, Doha, Qatar; ca Centro Hospitalar e Universitário São João, Porto, Portugal; cb Amsterdam UMC, Location VUmc, Amsterdam, Netherlands; cc Department of Hematology, CH Sud Francilien, Corbeil Essonnes, France; cd University Hospital Ostrava, Ostrava, Czech Republic; ce Weill Cornell Medicine, New York, United States; cf Laikon Hospital, Medical School, National and Kapodistrian University of Athens, Athens, Greece; cg KU Leuven, Leuven, Belgium; ch Hospital Universitario de Salamanca, Salamanca, Spain; ci University of Milan and Fondazione IRCCS Istituto Nazionale Dei Tumori, Milan, Italy; cj Division of Hematology, Department of Internal Medicine, University of Debrecen, Debrecen, Hungary; ck Department of Hematology, Hospital Universitario de Burgos, Burgos, Spain; cl Medizinische Klinik II, Klinikum Rechts der Isar, TU München, Munich, Germany; cm UOC Hematology, AORN Cardarelli, Naples, Italy; cn Aga Khan University, Karachi, Pakistan; co Hospital Universitario Lucus Augusti, Lugo, Spain; cp AOU Policlinico Rodolico San Marco, Catania, Italy; cq Hematology and Stem Cell Transplantation Unit, AOUC Policlinico, Bari, Italy; cr Hospital Universitario 12 de Octubre, Madrid, Spain; cs Clinical Microbiology Laboratory, Medical School, “Attikon” University General Hospital, National and Kapodistrian University of Athens, Athens, Greece; ct Hospital Universitario Infanta Leonor, Madrid, Spain; cu Istituto Oncologico della Svizzera Italiana, Bellinzona, Switzerland; cv Sultan Qaboos University Hospital, Muscat, Oman; cw Division of Infectious Diseases, ECMM Excellence Center for Clinical Mycology, Department of Internal Medicine, Medical University of Graz, Austria; cx BioTechMed, Graz, Austria; cy San Bortolo Hospital, Vicenza, Italy; cz University Hospital of Basel, Basel, Switzerland; da Aseer Central Hospital, Abha, Saudi Arabia; db Oncology Center Mansoura University, Mansoura, Egypt; dc Hopital Saint Louis, Paris, France; dd Hospital Rey Juan Carlos, Móstoles, Spain; de University of Kansas Medical Center, Kansas City, United States; df Department of Clinical Hematology, Montpellier University Hospital, IGMM UMR5535 CNRS, University of Montpellier, Montpellier, France; dg Azienda Ospedaliera Sant'Anna e San Sebastiano, Caserta, Italy; dh Northumbria Healthcare, Newcastle, United Kingdom; di University Hospital of Královské Vinohrady, Prague, Czech Republic; dj Department of Infectious Diseases and Clinical Microbiology, Faculty of Medicine Eskisehir Osmangazi University, Eskisehir, Turkey; dk Hospital Universitario Virgen del Puerto, Plasencia, Spain; dl Clinical Hematology Department, ICO-Hospital Germans Trias i Pujol, Josep Carreras Research Institute, Badalona, Spain; dm Cipto Mangunkusumo General Hospital, Jakarta, Indonesia; dn Hematology and Stem Cell Transplant Unit, Osperadiela University Pisana Company, Pisa, Italy; do “Sapienza” University of Rome, Rome, Italy; dp Service d'Hématologie Clinique et de Thérapie Cellulaire, Hôpital Saint Antoine, Assistance Publique-Hôpitaux de Paris, Sorbonne Université, Inserm UMRs 938, Paris, France; dq Instituto Nacional do Cancer, Rio de Janeiro, Brazil; dr Faculty of Medicine, University of Chile. Infectious Diseases Unity, Salvador Hospital of Santiago, Santiago de Chile, Chile; ds Azienda Ospedaliera “Ospedali Riuniti Villa Sofia-Cervello”, Palermo, Italy; dt Necker-Enfants Malades Hospitals, Paris, France; du Comenius University and National Cancer Institute, Bratislava, Slovakia; dv Universitätsklinikum Frankfurt am Main, Frankfurt am Main, Germany; dw Hospital Universitario de Cabueñes, Gijón, Spain; dx General Hospital of Thessaloniki “George Papanikolaou”, Thessaloniki, Greece; dy Medical University of Graz, Graz, Austria; dz Department of Mental Health and Public Medicine, University of Campania, Naples, Italy; ea Center for Radiology, University Clinical Center of Serbia, Belgrade, Serbia; eb University Hospital Pilsen, Pilsen, Czech Republic; ec Department of Medicine A for Hematology, Oncology and Pneumology, University Hospital Münster, Münster, Germany; ed Institute of Hematology and Blood Transfusion, Prague, Czech Republic; ee Department of Physiology, Faculty of Medicine, Masaryk University, Brno, Czech Republic; ef Hematology Unit, Fondazione Policlinico Universitario Agostino Gemelli - IRCCS, Rome, Italy; eg Hematology Unit, Università Cattolica del Sacro Cuore, Rome, Italy; eh University of Cologne, Faculty of Medicine and University Hospital Cologne, Clinical Trials Centre Cologne (ZKS Köln), Cologne, Germany; ei Université Paris-Saclay, UVSQ, Inserm, Équipe “Exposome et Hérédité”, CESP, Villejuif, France

**Keywords:** Vaccination, ICU, COVID-19, Haematological malignancy, Immunosuppression

## Abstract

**Background:**

The COVID-19 pandemic heightened risks for individuals with hematological malignancies due to compromised immune systems, leading to more severe outcomes and increased mortality. While interventions like vaccines, targeted antivirals, and monoclonal antibodies have been effective for the general population, their benefits for these patients may not be as pronounced.

**Methods:**

The EPICOVIDEHA registry (National Clinical Trials Identifier, NCT04733729) gathers COVID-19 data from hematological malignancy patients since the pandemic's start worldwide. It spans various global locations, allowing comprehensive analysis over the first three years (2020–2022).

**Findings:**

The EPICOVIDEHA registry collected data from January 2020 to December 2022, involving 8767 COVID-19 cases in hematological malignancy patients from 152 centers across 41 countries, with 42% being female. Over this period, there was a significant reduction in critical infections and an overall decrease in mortality from 29% to 4%. However, hospitalization, particularly in the ICU, remained associated with higher mortality rates. Factors contributing to increased mortality included age, multiple comorbidities, active malignancy at COVID-19 onset, pulmonary symptoms, and hospitalization. On the positive side, vaccination with one to two doses or three or more doses, as well as encountering COVID-19 in 2022, were associated with improved survival.

**Interpretation:**

Patients with hematological malignancies still face elevated risks, despite reductions in critical infections and overall mortality rates over time. Hospitalization, especially in ICUs, remains a significant concern. The study underscores the importance of vaccination and the timing of COVID-19 exposure in 2022 for enhanced survival in this patient group. Ongoing monitoring and targeted interventions are essential to support this vulnerable population, emphasizing the critical role of timely diagnosis and prompt treatment in preventing severe COVID-19 cases.

**Funding:**

Not applicable.

**Research in context**

**Evidence before this study**

Prior to this study, existing research had shed light on the risks posed by COVID-19 to hematological malignancy patients. Historical data from past pandemics, indicated that individuals with hematological malignancies were particularly susceptible to severe disease and elevated mortality rates. Early in the COVID-19 pandemic, hematological malignancy patients faced substantial challenges, with higher hospitalization rates and poorer outcomes. Furthermore, the emergence of SARS-CoV-2 variants and potential waning vaccine efficacy raised concerns about the ongoing vulnerability of this population.

**Added value of this study**

This study advances our understanding of COVID-19 outcomes in hematological malignancy patients by analyzing data spanning from 2020 to 2022, encompassing the transition from pandemic to epidemic phases. Leveraging a vast dataset from 152 institutions across 41 countries through the EPICOVIDEHA registry, this research provides a comprehensive examination of risk factors, disease severity, and mortality rates. Notably, it highlights the critical role of vaccination and early therapeutic interventions, including targeted antivirals and monoclonal antibodies, in improving prognosis. The study's insights into the evolving landscape of hematological malignancies and the effectiveness of various treatment strategies contribute vital knowledge to guide clinical decision-making.

**Implications of all the available evidence**

Collectively, the available evidence, including findings from this study, underscores the ongoing challenges faced by hematological malignancy patients in managing COVID-19. Despite the transition from pandemic to epidemic phases, this population remains at risk due to their compromised immune systems. The implications of this evidence emphasize the need for continued vigilance, personalized care, and the optimization of vaccination strategies, including booster doses. Additionally, the study's findings underscore the significance of early therapeutic interventions and tailored treatment approaches based on vaccination status and disease severity. As the COVID-19 situation continues to evolve, healthcare providers must remain adaptable, leveraging the latest evidence to deliver the highest quality care to hematological malignancy patients. It is worth noting that, even though three years have elapsed since the onset of the pandemic, randomized studies in hematology patients with COVID-19 are still lacking. Therefore, it is crucial to exercise caution with data interpretation as there may be aspects where we continue to make errors.

## Introduction

Since the emergence of the coronavirus disease 2019 (COVID-19) pandemic, hematological malignancy patients have faced substantial risks, being highly vulnerable to contracting severe acute respiratory syndrome coronavirus type 2 (SARS-CoV-2) and experiencing severe outcomes, including elevated hospitalization rates and increased fatality.^1^ COVID-19 pandemic reminded us the history of other pandemics that happened in the last century. In accordance with those, following the pandemic phase, we are facing now an epidemic one, characterized by less morbidity and severity of the virus in the overall population.^2^^,^^3^ However, hematological malignancy patients still remain at risk of severe COVID-19. Despite advancements in medical interventions, such as the introduction of anti-SARS-CoV-2 vaccines,^4^ targeted antivirals,^5^ and monoclonal antibodies,^6^ which have shown promising results in reducing the impact of COVID-19 on otherwise healthy individuals, the same level of effectiveness cannot always be guaranteed for patients with hematological malignancies.

This discrepancy can be attributed to their compromised immune system, due to both intrinsic disease biology and immunosuppressive treatments, rendering them less responsive to vaccines and more susceptible to severe COVID-19.^7^, ^8^, ^9^, ^10^, ^11^, ^12^, ^13^, ^14^, ^15^, ^16^ As such, understanding the factors influencing COVID-19 outcomes in this patient population becomes paramount in improving their overall management and care. Given the unique challenges faced by hematological malignancy patients in managing COVID-19, identifying risk factors and protective factors can significantly inform clinical decision-making and enhance patient outcomes. In response to the onset of the pandemic, researchers globally engaged in a range of analyses across various research levels. These encompassed detailed case reports and case series, extensive observational studies to derive broader insights, and comprehensive literature reviews and meta-analyses to synthesize existing knowledge. These combined efforts significantly enriched our understanding of the pandemic's multifaceted implications.^17^ However, within the context of individuals dealing with hematological malignancies, several notable gaps in understanding remain; while existing research has shed light on specific aspects of the interaction between COVID-19 and these malignancies, important questions still await exploration.^14^^,^^18^ The intricate interplay between the infection, its progression, and the management strategies employed within this patient population necessitates further investigation.

The present study was designed to thoroughly investigate the outcomes and potential risk factors linked to COVID-19 in patients with hematological malignancies from 2020 to 2022, describing not only a transversal picture, but also the pandemic trajectory over those years in this peculiar patient category. Specifically, we aimed to assess the influence of diverse demographic characteristics, comorbidities, underlying malignancies, and vaccination status on mortality rates and disease severity.

## Methods

### Study design, patients and procedures

Participating institutions (n = 152) from 41 countries (Supplementary Fig. S1) documented COVID-19 episodes diagnosed between January 1, 2020, and December 31, 2022 in patients with hematological malignancies in the EPICOVIDEHA registry, accessible at www.clinicalsurveys.net (EFS Fall 2022, TIVIAN, Cologne, Germany), including both already described^2^^,^^7^^,^^8^^,^^10^^,^^11^^,^^13^^,^^19^, ^20^, ^21^, ^22^, ^23^, ^24^, ^25^, ^26^, ^27^, ^28^, ^29^, ^30^, ^31^, ^32^, ^33^ and undescribed patients. EPICOVIDEHA (National Clinical Trials Identifier, NCT04733729) is an international open web-based registry dedicated to adult hematological malignancy patients infected with SARS-CoV-2, set by part of the European Hematology Association Specialized Working Group (EHA-SWG) Infections in Hematology.^30^ The registry was approved by the local ethics committee of the Fondazione Policlinico Universitario Agostino Gemelli, IRCCS, Università Cattolica del Sacro Cuore of Rome, Italy (Study ID: 3226), and was also approved by the respective local ethics committees of each participating institution if required.

To be included in the study, patients had to meet certain criteria: they must have had an active hematological malignancy within the past 5 years before their COVID-19 diagnosis, be aged 18 years or older, have a laboratory-confirmed SARS-CoV-2 infection (either by polymerase chain reaction [PCR] or rapid antigen test kits), and have received a COVID-19 diagnosis between January 1, 2020, and December 31, 2022. Patients with solid tumors or non-malignant hematological disorders, including aplastic anemia, children, patients off-therapy or cured for longer than 5 years before COVID-19 diagnosis or those with only an imaging procedure-based COVID-19 diagnosis were excluded from analysis. Data collected for each patient encompassed baseline conditions before COVID-19, such as age, biological sex, hematological malignancy status at COVID-19 diagnosis, and COVID-19 predisposing factors. Additionally, details regarding hematological malignancy clinical management (i.e., malignancy status, type of last malignancy treatment immediately before COVID-19 diagnosis), and COVID-19 diagnosis, symptomatology, prophylaxis and treatments (including type number of anti-SARS-CoV-2 vaccines at COVID-19 onset and anti-SARS-CoV-2 treatment strategies), and outcomes (including mortality and last follow-up date) were recorded. The status of hematological malignancy at COVID-19 onset was categorized as active (onset and refractory/resistant), stable disease, or controlled (complete and partial response) based on reports from the participating institutions. Additionally, patients were graded based on the severity of their COVID-19 episodes as asymptomatic, mild, severe and critical as previously defined.^34^

To ensure data coherence and completeness, all patients were included in a validation process conducted by experts in hematological malignancy and infectious diseases. During this process, efforts were made to reduce data missing completely at random by contacting contributors to resolve pending queries. This validation process was crucial for maintaining the reliability and integrity of the data collected from the EPICOVIDEHA registry.^30^ If a certain variable had missing data among valid cases and was included in regression analyses, those patients were also excluded from the analysis. Nevertheless, sensitivity analyses were performed applying the following methods: series mean, linear interpolation, and linear trend at point.

### Study objectives

The primary objective of the study was to analyze the epidemiology and outcomes of hematological malignancy patients affected by COVID-19 during the period 2020–2022. Secondary objectives included estimating the relative frequency of disease severity, evaluating the relative frequency of intensive care unit (ICU) admission among participating patients, assessing the overall case-fatality rate, exploring the effect of cancer treatment phase on patient outcomes, investigating the impact of vaccine doses administered on patient outcomes, and studying the effect of COVID-19 treatment on patient outcomes.

### Statistical analysis

An a priori sample size calculation was not performed as the study was exploratory in nature. Data from participating institutions were summarized using frequencies and percentages for categorical variables, and median, interquartile range (IQR), and absolute range for continuous variables. A univariable Cox regression model was employed to analyze variables suspected to influence the mortality of hematological malignancy patients with COVID-19. Variables with a p value ≤ 0.1 were considered for multivariable analysis, after confirming their clinical relevance. The multivariable Cox regression model was calculated using the Wald backward method. The multivariable Cox regression model was constructed using backward elimination with the Wald test for variable selection. Exclusion criteria were established a priori, guided by a predetermined statistical significance threshold (p value ≤ 0.05). This approach retained variables that significantly contributed to the model. Mortality was analyzed using Kaplan–Meier survival plots, and a log-rank test was used to compare the survival probability of patients included in different models. The Cox proportional hazards model was applied to analyze variables influencing the mortality of hematological malignancy patients with COVID-19. Assumptions of the model were rigorously assessed: tests for proportional hazards (Schoenfeld residual test) were conducted to verify the assumption of hazard functions' proportionality across strata, and scatterplots and diagnostic tools were utilized to confirm the linearity of the relationship between the log hazard and each covariate (Plot martingale residuals). We employed Cox regression analysis to assess the association between various covariates and the survival time of patients. The following variables were included in the multivariable Cox regression model: sex, age, comorbidities, baseline malignancy, malignancy status at COVID-19 diagnosis, vaccine doses before COVID-19, COVID-19 diagnosis time periods, COVID-19 symptoms at onset, and stay during COVID-19 episode. To account for the potential issue of multiple comparisons, we applied the Bonferroni correction. This correction involved adjusting the significance level for each individual test to maintain an overall family-wise error rate. Specifically, we divided the desired overall significance level (p = 0.05) by the number of tests conducted. The adjusted significance level was calculated as 0.05/9, where 9 represents the number of variables tested. Therefore, p-values less than 0.0056 were considered statistically significant after Bonferroni correction. Additionally, we reported Hazard Ratios (HR) and 95% Confidence Intervals (CI) to quantify the strength and precision of associations. A p value ≤ 0.05 was considered statistically significant. The statistical analyses were conducted using SPSS version 25.0 (SPSS, IBM Corp, Chicago, IL, United States).

### Ethics statement

The central ethics committee is at Fondazione Policlinico Universitario Agostino Gemelli - IRCCS, Università Cattolica del Sacro Cuore of Rome, Italy (Study ID: 3226). Additionally, each participating institution may also have a local approval for the research initiative as appropriate. The anonymized data that do not contain any personally identifiable information from any sources implies that the informed consent is not applicable.

### Role of the funding source

This research did not receive any funding. JSG, FM, LP, and OAC had access to and verified all raw data sets and made the decision to submit the manuscript. The corresponding author can provide the data supporting the findings of this study upon a reasonable request.

## Results

Out of the 9416 COVID-19 episodes diagnosed between January 1, 2020, and December 31, 2022 and documented in the EPICOVIDEHA registry, 8767 were set valid and included to analysis. Out of the 649 cases set invalid, 179 patients had non-malignant hematological disorders or solid tumors, 135 patients were excluded as their diagnosis of COVID-19 was based on clinical criteria rather than laboratory confirmation, 93 patients had hematological malignancies diagnosed longer than six months after contracting COVID-19, 87 patients had been off therapy for more than 5 years, 86 cases were removed due to incomplete information, 55 patients were below the age of 18, and 14 cases had double entries.

**Fig. 1:**
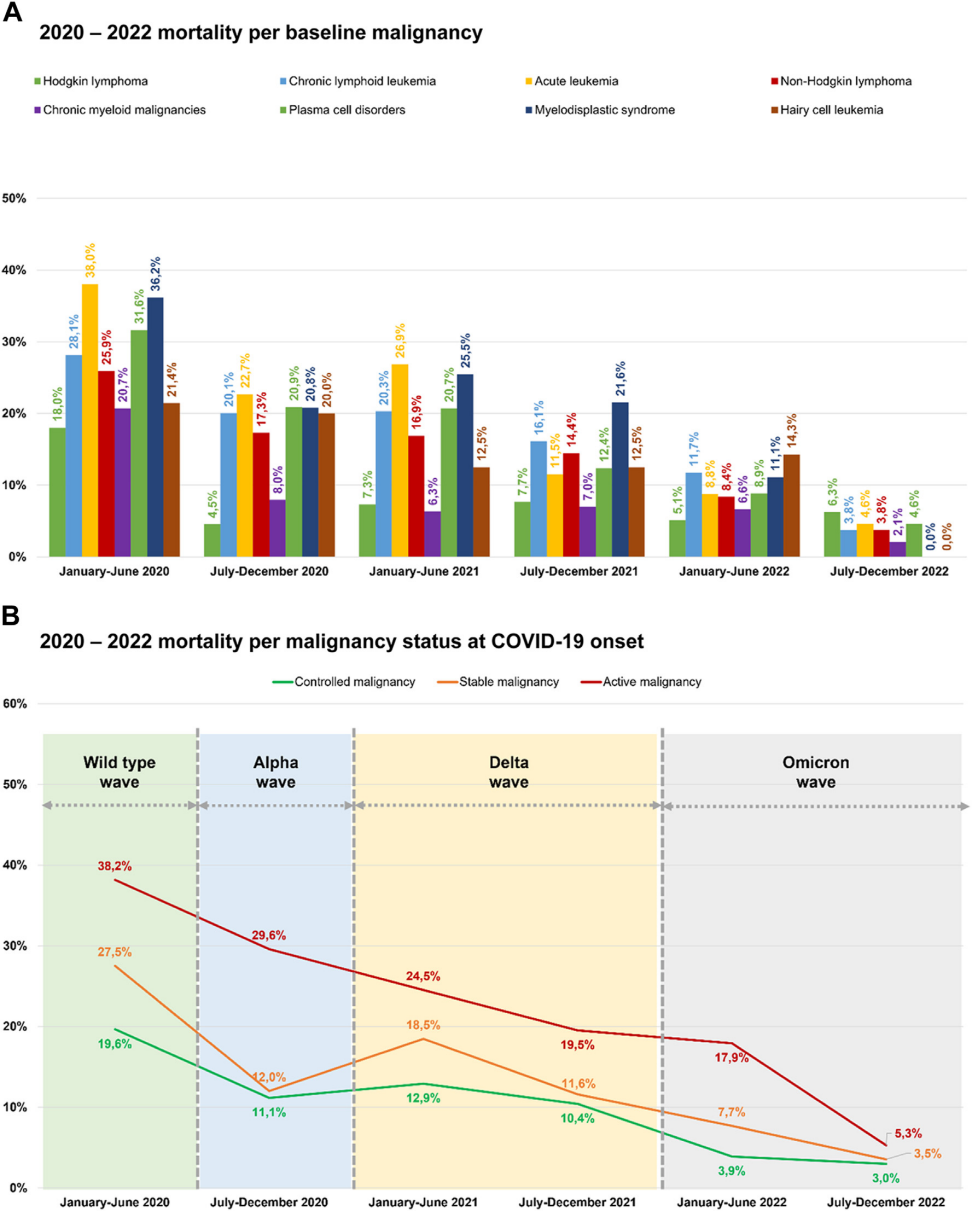

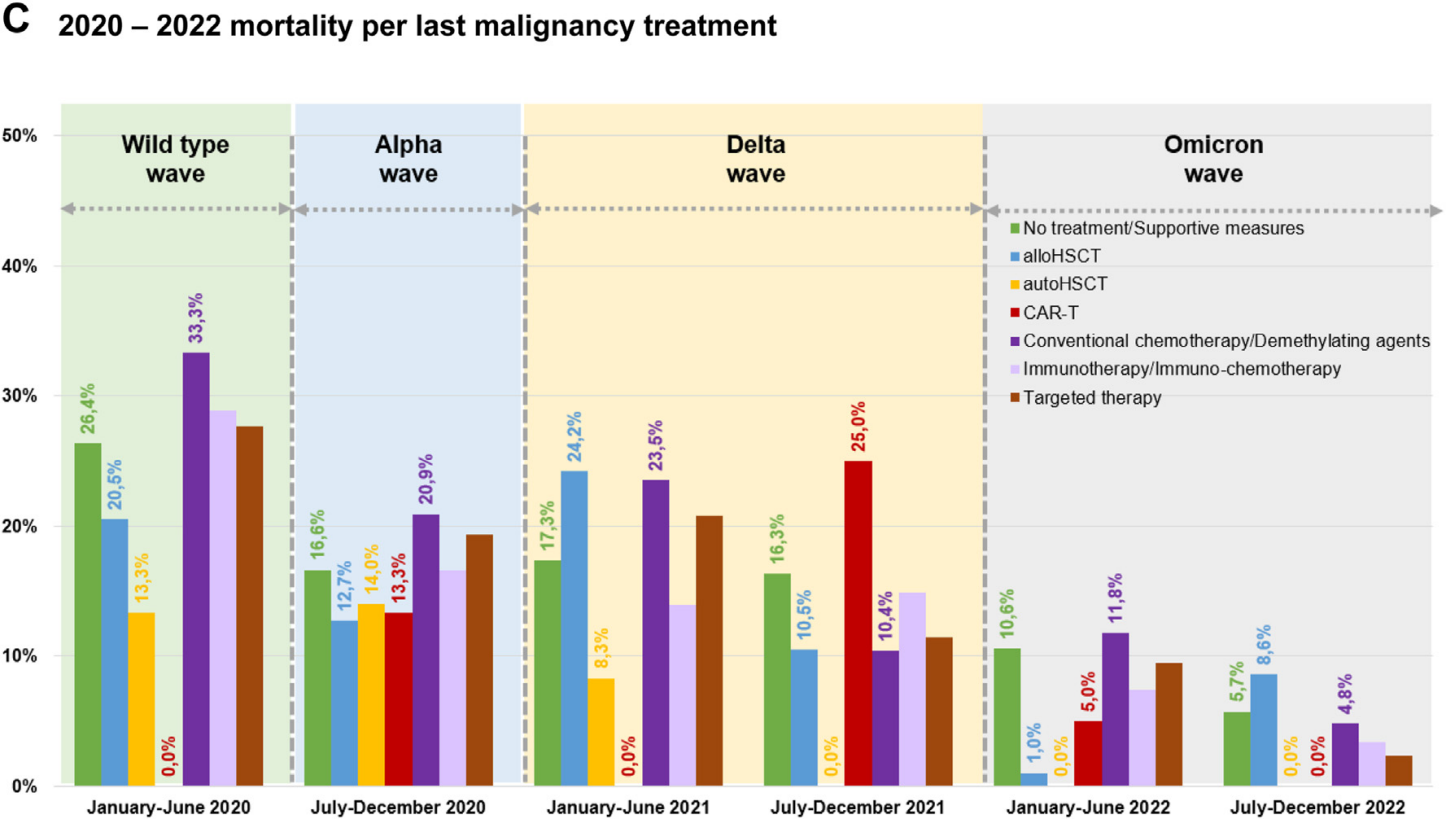
EPICOVIDEHA 2020–2022 patients: mortality per baseline malignancy, malignancy status at COVID-19 onset, and last malignancy treatment. A) 2020–2022 mortality per baseline malignancy. B) 2020–2022 mortality per malignancy status at COVID-19 onset. C) 2020–2022 mortality per last malignancy treatmentAllo, allogeneic; auto, autologous; CAR-T, chimeric antigen T cell receptors; HSCT, hematopoietic stem cell transplantation.

The overall sample included 8767 patients, with nearly 60% (n = 5119) being male. The median age was 65 years (IQR: 54–75); the youngest patient was 18, and the oldest was 106, with majority of patients (79.6%, n = 6975) older than 50 years. Age groups showed statistically significant differences in mortality rates (p < 0.0001) (Table 1, Supplementary Tables S1–S6). Regarding comorbidities, 70.8% (n = 6205) of the patients had 0-1 comorbidities, with a significantly lower mortality rate as compared to those with 2 or more comorbidities (p < 0.0001). The most common comorbidities observed were chronic cardiopathy (35.3%, n = 3095), diabetes mellitus (14.5%, n = 1268), and chronic pulmonary disease (13.2%, n = 1157) (Supplementary Fig. S2A). As for the underlying malignancies, non-Hodgkin lymphoma (31.2%, n = 2731) and plasma cells disorders (17.6%, n = 1546) were the most prevalent (Supplementary Fig. S2B). However, myelodysplastic syndrome (21.2%, n = 121/572), acute leukemia (19.0%, n = 280/1475), and chronic lymphoid leukemia (18.1%, n = 210/1163) exhibited the highest mortality rates, all of which were statistically significant (p < 0.0001) (Fig. 1A). Almost half of the patients (47.1%, n = 4131) had controlled malignancy. Among them, patients with controlled malignancy (10.2%, n = 420/4131) and stable malignancy (13.6%, n = 230/1688) experienced lower mortality rates compared to those with active malignancy (25.5%, n = 676/2646), p < 0.0001 (Fig. 1B, Table 1, Supplementary Tables S1–S6). Regarding the vaccination status against SARS-CoV-2, 64.5% (n = 5658) of patients were unvaccinated as they were diagnosed with COVID-19 before the availability of the first vaccines, and this cohort exhibited the highest mortality rate at 20.8%.

**Table 1:** Characteristics of EPICOVIDEHA 2020–2022 patients.

	Total		Alive		Dead		p value
	n	%	n	%	n	%	
**Sex**							0.08
Female	3648	41.6	3089	84.7	559	15.3	
Male	5119	58.4	4263	83.3	856	16.7	
**Age**	65 (54–75) [18–106]		64 (52–73) [18–97]		73 (63–80) [18–106]		<0.0001
18–25 years old	268	3.1	244	91.0	24	9.0	
26–50 years old	1524	17.4	1411	92.6	113	7.4	
51–69 years old	3517	40.1	3094	88.0	423	12.0	
>69 years old	3458	39.4	2603	75.3	855	24.7	
**Comorbidities at COVID-19 onset**							
0-1 comorbidities	6205	70.8	5401	87.0	804	13.0	<0.0001
2+ comorbidities	2562	29.2	1951	76.2	611	23.8	
Chronic cardiopathy	3095	35.3	2422	78.3	673	21.7	<0.0001
Chronic pulmonary disease	1157	13.2	892	77.1	265	22.9	<0.0001
Diabetes mellitus	1268	14.5	979	77.2	289	22.8	<0.0001
Liver disease	355	4.0	267	75.2	88	24.8	<0.0001
Renal impairment	648	7.4	456	70.4	192	29.6	<0.0001
Smoking history	978	11.2	781	79.9	197	20.1	0.00031
**Baseline hematological malignancy**							<0.0001
Hodgkin lymphoma	354	4.0	328	92.7	26	7.3	
Chronic lymphoid leukemia	1163	13.3	953	81.9	210	18.1	
Acute leukemia	1475	16.8	0	0.0	280	19.0	
Acute myeloid leukemia	1060	12.1	824	77.7	236	22.3	
Acute lymphoid leukemia	415	4.7	371	89.4	44	10.6	
Non-Hodgkin lymphoma	2731	31.2	2315	84.8	416	15.2	
Chronic myeloid malignancies	867	9.9	780	90.0	87	10.0	
Chronic myeloid leukemia	334	3.8	313	93.7	21	6.3	
Essential thrombocythemia	142	1.6	131	92.3	11	7.7	
Myelofibrosis	233	2.7	190	81.5	43	18.5	
Polycythemia vera	134	1.5	123	91.8	11	8.2	
Systemic mastocytosis	24	0.3	23	95.8	1	4.2	
Plasma cell disorders	1546	17.6	1281	82.9	265	17.1	
Multiple myeloma	1513	17.3	1249	82.6	264	17.4	
Amyloid light-chain amyloidosis	33	0.4	32	97.0	1	3.0	
Myelodysplastic syndrome	572	6.5	451	78.8	121	21.2	
Hairy cell leukemia	59	0.7	49	83.1	10	16.9	
**Baseline hematological malignancy status at COVID-19 onset**							<0.0001
Controlled malignancy	4131	47.1	3711	89.8	420	10.2	
Complete remission	2717	31.0	2502	92.1	215	7.9	
Partial remission	1414	16.1	1209	85.5	205	14.5	
Stable malignancy	1688	19.3	1458	86.4	230	13.6	
Active malignancy	2646	30.2	1970	74.5	676	25.5	
Onset	1553	17.7	1195	76.9	358	23.1	
Refractory/Resistant	1093	12.5	775	70.9	318	29.1	
Unknown	302	3.4	213	70.5	89	29.5	
**SARS-CoV-2 vaccination at COVID-19 onset**							<0.0001
0 doses	5658	64.5	4480	79.2	1178	20.8	
1–2 doses	1328	15.1	1192	89.8	136	10.2	
1 dose	214	2.4	192	89.7	22	10.3	
2 doses	1114	12.7	1000	89.8	114	10.2	
3+ doses	1781	20.3	1680	94.3	101	5.7	
3 doses	1484	16.9	1391	93.7	93	6.3	
4 doses	274	3.1	266	97.1	8	2.9	
5 doses	23	0.3	23	100.0	0	0.0	
**COVID-19 symptoms at onset**							<0.0001
Screening	1787	20.4	1605	89.8	182	10.2	
Extrapulmonary only	1717	19.6	1554	90.5	163	9.5	
Extrapulmonary + pulmonary	2237	25.5	1781	79.6	456	20.4	
Pulmonary only	3026	34.5	2412	79.7	614	20.3	
**COVID-19-episode severity**							<0.0001
Asymptomatic	1546	17.6	1442	93.3	104	6.7	
Mild infection	1651	18.8	1545	93.6	106	6.4	
Severe infection	4291	48.9	3655	85.2	636	14.8	
Critical infection	1279	14.6	710	55.5	569	44.5	
**Stay during COVID-19**							<0.0001
Home	3084	35.2	3056	99.1	28	0.9	
Hospital no ICU	4404	50.2	3586	81.4	818	18.6	
Hospital ICU	1279	14.6	710	55.5	569	44.5	
**Mortality, d30**	1415	16.1	0	0.0	1415	100.0	
Reason for mortality							
COVID-19	904	10.3	0	0.0	904	63.9	
COVID-19 + hematological malignancy	367	4.2	0	0.0	367	25.9	
Hematological malignancies ± other reasons	144	1.6	0	0.0	144	10.2	

COVID-19, coronavirus disease 2019; ICU, intensive care unit; SARS-CoV-2, severe acute respiratory syndrome coronavirus 2.

**Fig. 2:**
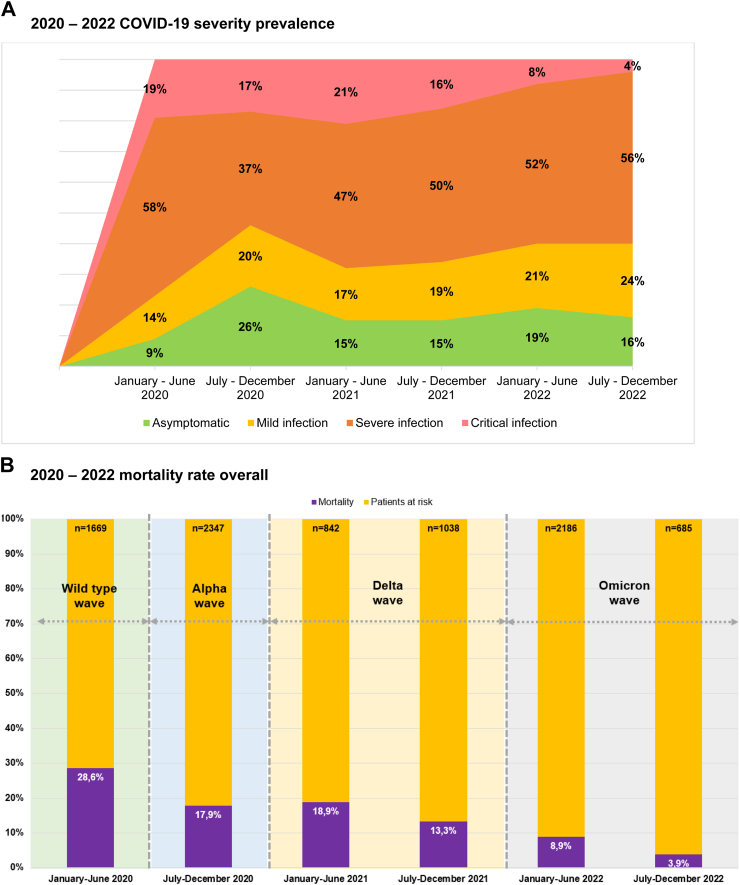

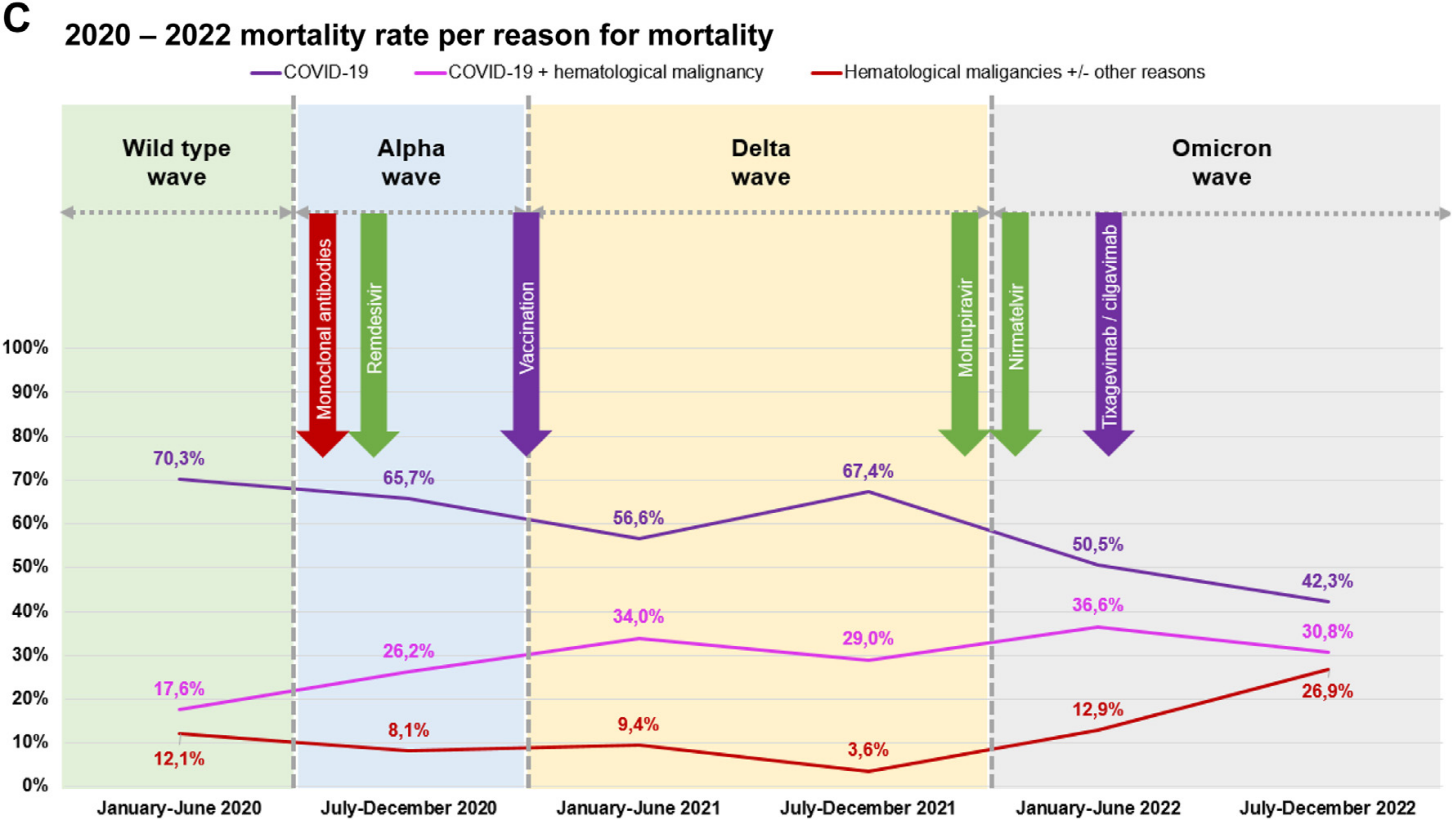
EPICOVIDEHA 2020–2022 patients: COVID-19 severity prevalence and mortality rate, overall mortality rate, mortality per malignancy status at COVID-19 onset, and mortality per reason for mortality. A) 2020–2022 COVID-19 severity prevalence. B) 2020–2022 mortality rate overall. C) 2020–2022 mortality rate per reason for mortality. COVID-19, coronavirus disease 2019. ∗Arrows in this figure indicate the date when the respective medical product was made available for the very first time in the world. This is applicable to some of the contributing institutions, not to them all.

Mortality rates decreased with an increasing number of anti-SARS-CoV-2 vaccine doses administered before COVID-19 diagnosis (1 dose: 10.3%, n = 22/214; 5 doses: 0.0%, n = 0/23), with a statistically significant difference (p < 0.001) (Table 1, Supplementary Tables S1–S6). Six in ten patients (60.0%, n = 5263) experienced pulmonary symptoms at the onset of COVID-19. Overall, 36.5% (n = 3197) of patients had asymptomatic or mild infection (Fig. 2A). One third of the patients (35.2%, n = 3084) were managed at home, while 64.8% (n = 5683) were admitted to the hospital at any point during COVID-19. Among the hospitalized patients, 16.1% required admission to the ICU (Table 1, Supplementary Tables S1–S6). The overall 30-day mortality rate was 16.1% (n = 1415/8767, Fig. 2B), with COVID-19 being associated with mortality in 89.8% of these cases (Fig. 2C, Table 1, Supplementary Tables S1–S6). The following factors linked to increased mortality: a) older age (aHR 1.037, 95% CI 1.032–1.042, p < 0.0001), b) 2 or more comorbidities at COVID-19 onset (aHR 1.244, 95% CI 1.117–1.386, p < 0.0001), c) active malignancy (aHR 1.832, 95% CI 1.617–2.075, p < 0.0001), d) pulmonary symptoms at COVID-19 onset, either alone (aHR 1.299, 95% CI 1.095–1.541, p = 0.0026) or in combination with extrapulmonary symptoms (aHR 1.168, 95% CI 0.978–1.396, p = 0.08), and e) hospitalized patients without ICU (aHR 12.767, 95% CI 8.723–18.684, p < 0.0001) and patients admitted to the ICU (aHR 33.684, 95% CI 22.915–49.514, p < 0.0001). On the other hand, certain factors were associated with reduced mortality, such as: a) vaccination with 1–2 doses (aHR 0.681, 95% CI 0.546–0.850, p < 0.00066) or with 3 or more (aHR 0.451, 95% CI 0.35–0.582, p < 0.0001), b) COVID-19 diagnosis later in the pandemic, specifically between July–December 2020 (aHR 0.802, 95% CI 0.701–0.917, p < 0.0012), January–June 2021 (aHR 0.796, 95% CI 0.662–0.958, p = 0.015) and July–December 2022 (aHR 0.427, 95% CI 0.282–0.647, p < 0.000059) (Table 2). Sensitivity analyses were conducted to address the absence of data on observation days from certain patients. The outcomes obtained showed consistency and endorsed identical variables as either risk or protective factors, in alignment with the aforementioned paragraph. Comprehensive results can be found in Supplementary Tables S7–S9.

**Table 2:** Factors associated with mortality in EPICOVIDEHA 2020–2022 patients.

	Univariable				Multivariable			
	p value	HR	95% CI		p value	HR	95% CI	
			Lower	Upper			Lower	Upper
**Sex**								
Female	–	–	–	–	–	–	–	–
Male	0.08	1.100	0.988	1.223	0.99	1.001	0.899	1.115
**Age**	<0.0001	1.037	1.033	1.041	<0.0001	1.037	1.032	1.042
**Comorbidities**								
0–1 comorbidities	–	–	–	–	–	–	–	–
2+ comorbidities	<0.0001	1.917	1.726	2.130	<0.0001	1.244	1.117	1.386
**Baseline malignancy**								
Hodgkin lymphoma	–	–	–	–	–	–	–	–
Chronic lymphoid leukemia	<0.0001	2.638	1.755	3.965	0.52	0.874	0.578	1.32
Acute leukemia	<0.0001	2.818	1.885	4.211	0.08	1.431	0.956	2.141
Non-Hodgkin lymphoma	0.00015	2.152	1.448	3.198	0.49	0.868	0.583	1.293
Chronic myeloid malignancies	0.12	1.417	0.914	2.196	0.20	0.747	0.479	1.163
Plasma cell disorders	<0.0001	2.503	1.673	3.744	0.71	1.08	0.72	1.621
Myelodysplastic syndrome	<0.0001	3.163	2.071	4.832	0.98	0.985	0.64	1.517
Hairy cell leukemia	0.018	2.416	1.165	5.011	0.51	0.781	0.376	1.623
**Malignancy status at COVID-19 diagnosis**								
Controlled malignancy	–	–	–	–	–	–	–	–
Stable malignancy	<0.0001	1.394	1.187	1.637	0.21	1.115	0.94	1.321
Active malignancy	<0.0001	2.731	2.418	3.085	<0.0001	1.832	1.617	2.075
**Vaccine doses before COVID-19**								
0 doses	–	–	–	–	–	–	–	–
1–2 doses	<0.0001	0.503	0.421	0.601	0.00067	0.681	0.546	0.850
3+ doses	<0.0001	0.286	0.233	0.350	<0.0001	0.451	0.35	0.582
**COVID-19 diagnosis**								
January–June 2020	–	–	–	–	–	–	–	–
July–December 2020	<0.0001	0.577	0.506	0.657	0.0012	0.802	0.701	0.917
January–June 2021	<0.0001	0.618	0.517	0.740	0.015	0.796	0.662	0.958
July–December 2021	<0.0001	0.470	0.388	0.567	0.59	0.938	0.745	1.18
January–June 2022	<0.0001	0.322	0.273	0.381	0.08	0.833	0.679	1.021
July–December 2022	<0.0001	0.132	0.089	0.197	<0.0001	0.427	0.282	0.647
**COVID-19 symptoms at onset**								
Screening	–	–	–	–	–	–	–	–
Extrapulmonary only	0.49	0.928	0.751	1.146	0.13	0.848	0.685	1.049
Extrapulmonary + pulmonary	<0.0001	2.131	1.794	2.530	0.09	1.168	0.978	1.396
Pulmonary only	<0.0001	2.149	1.821	2.535	0.0026	1.299	1.095	1.541
**Stay during COVID-19 episode**								
Home	–	–	–	–	–	–	–	–
Hospital, no ICU	<0.0001	22.360	15.342	32.589	<0.0001	12.767	8.723	18.684
Hospital, ICU	<0.0001	57.020	39.015	83.335	<0.0001	33.684	22.915	49.514

COVID-19, coronavirus disease 2019; HR, hazard ratio; ICU, intensive care unit.

After applying the Bonferroni correction, variables with a p < 0.0056 were considered as statistically significant. The variables age, comorbidities, malignancy status at COVID-19 diagnosis, vaccine doses before COVID-19, COVID-19 diagnosis (item July–December 2020), COVID-19 symptoms at onset, and stay during COVID-19 episode remain significant. Other variables do not reach the adjusted significance level.

To address missing data, sensitivity analyses were performed. In Supplementary Table S7 missing values in *Days from COVID-19 diagnosis* were input with the series mean method, in Supplementary Table S8 missing values in *Days from COVID-19 diagnosis* were input with the linear interpolation method, and in Supplementary Table S9 missing values in *Days from COVID-19 diagnosis* were input with the linear trend at point method.

Hazard function proportionality over the time t and lineal relationship between the log hazard and each covariate in the model depicted in the table have been analyzed and are presented in Supplementary Figs. S4–S7. Supplementary Fig. S4 presents the proportional hazard evaluation of the variables presented in Table 2. p values for the Schoenfeld residual test are as follows: age p = 0.09 (proportional), baseline malignancy p = 0.0057 (non-proportional), month of COVID-19 diagnosis p < 0.0001 (non-proportional), stay during COVID-19 episodes p < 0.0001 (non-proportional), vaccine doses before COVID-19 p = 0.0027 (non-proportional), comorbidities p = 0.88 (proportional), COVID-19 symptoms at onset p = 0.0018 (non-proportional), sex p = 0.68 (proportional), and malignancy status at COVID-19 diagnosis p = 0.11 (proportional). Plot martingale residuals are presented in Supplementary Fig. S5. Sensitivity analyses were performed for the proportional hazard evaluation, reducing the answer categories presented in Table 2. These are shown in Supplementary Fig. S6. p values for the Schoenfeld residual test are as follows: age p = 0.09 (proportional), baseline malignancy p = 0.0054 (non-proportional), month of COVID-19 diagnosis p < 0.0001 (non-proportional), stay during COVID-19 episodes p < 0.0001 (non-proportional), vaccine doses before COVID-19 p = 0.00071 (non-proportional), comorbidities p = 0.88 (proportional), COVID-19 symptoms at onset p = 0.00086 (non-proportional), sex p = 0.69 (proportional), and malignancy status at COVID-19 diagnosis p = 0.11 (proportional). Plot martingale residuals are presented in Supplementary Fig. S7.

### Changes over time

During the study period from 2020 to 2022, the patient distribution in terms of sex, age group, number of comorbidities (Supplementary Fig. S2A), and malignancy status at COVID-19 onset or last malignancy treatment remained stable (Supplementary Fig. S2C). Non-Hodgkin lymphoma exhibited stability, while there was an observed rise in the prevalence of plasma cell disorders, along with acute leukemia. Conversely, myelodysplastic syndrome and chronic myeloid malignancies demonstrated a decline (Supplementary Fig. S2B). Moreover, there was an increase in the number of vaccinated patients, those not requiring hospitalization and managing their condition at home during the COVID-19 episode (Supplementary Tables S1–S6). Alongside this, there was a notable reduction in the severity of COVID-19 cases (Fig. 2A, Supplementary Tables S1–S6).

A decline in COVID-19-related mortality across all severity degrees was observed (Supplementary Tables S1–S6), as well as in all-cause mortality (Fig. 2B). Furthermore, this decrease in mortality was evident when considering type of baseline malignancy (Fig. 2A), malignancy treatment (Fig. 1C), malignancy status at COVID-19 onset (Fig. 1B), and the cause of death (Fig. 2C). Among causes of death there was a decrease in the influence of COVID-19-related factors and a corresponding increase in the impact of hematological malignancy. This reduction in mortality rates was evident in the overall analysis but was more limited for in-hospital and critical patients.

**Fig. 3:**
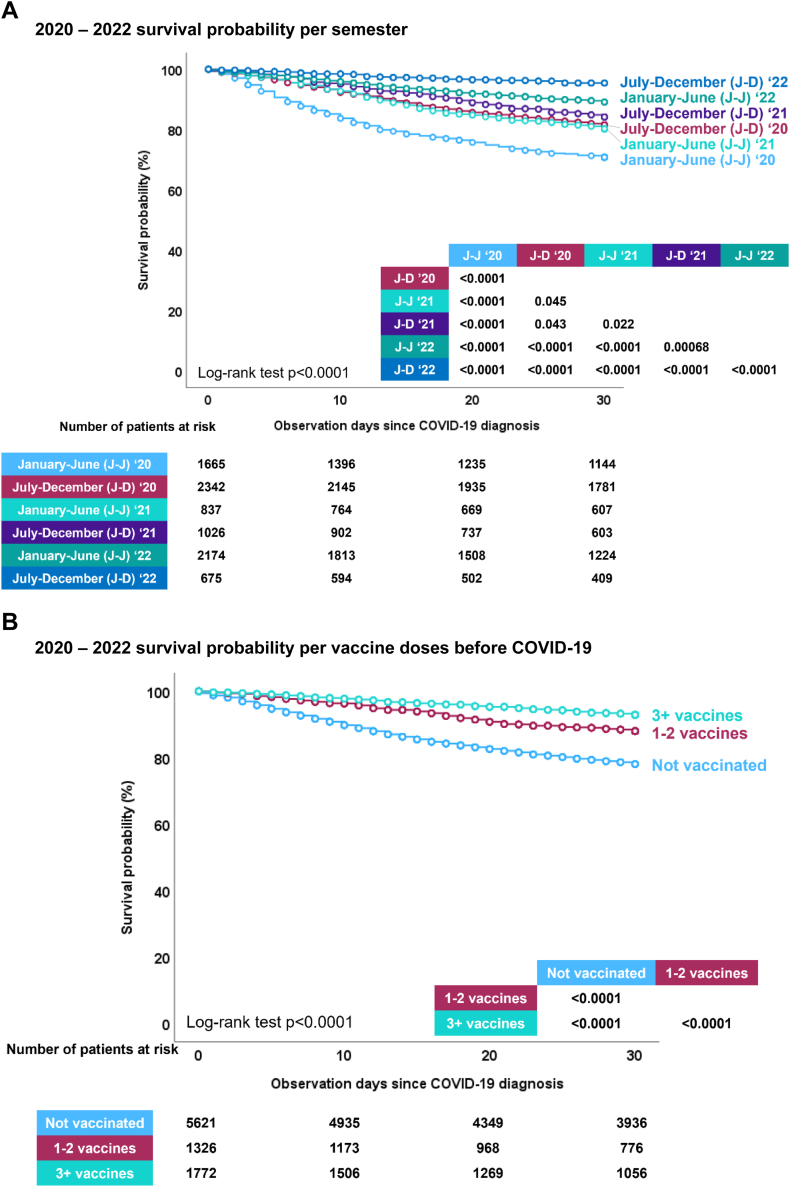

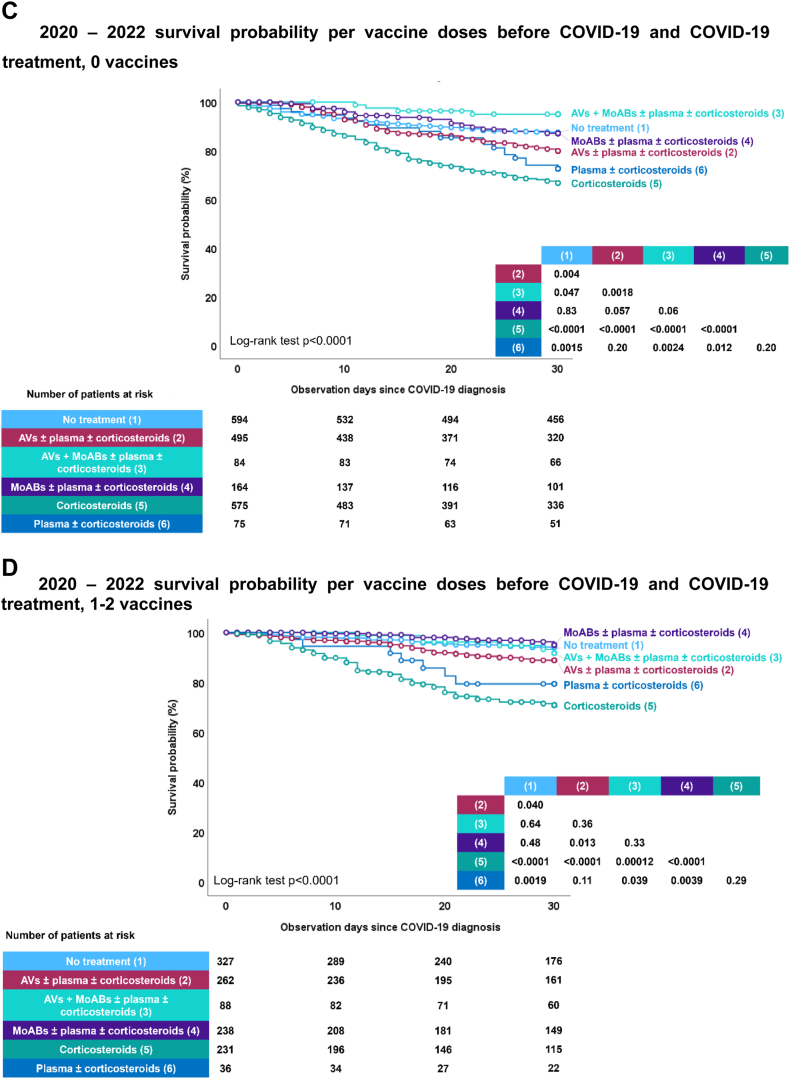

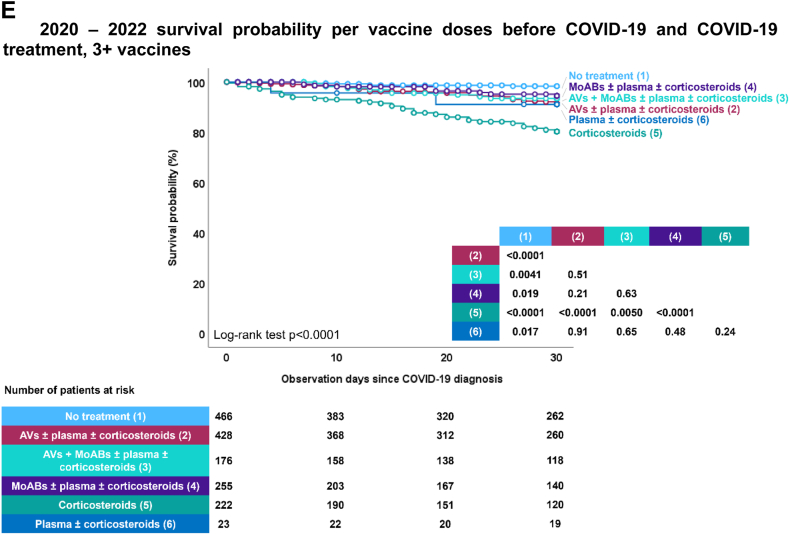
EPICOVIDEHA 2020–2022 patients: survival probability per diagnosis semester, vaccination status, and COVID-19 treatment. A) 2020–2022 survival probability per semester. This figure has been replicated with the survival probability ranging from 70% to 100% in Supplementary Fig. S8. B) 2020–2022 survival probability per vaccine doses before COVID-19. This figure has been replicated with the survival probability ranging from 70% to 100% in Supplementary Fig. S9. C) 2020–2022 survival probability per vaccine doses before COVID-19 and COVID-19 treatment, 0 vaccines. AVs, antivirals; MoABs, monoclonal antibodies. ∗ No treatment includes patients that did not need to receive treatment mainly due to lack of symptoms. This figure has been replicated with the survival probability ranging from 70% to 100% in Supplementary Fig. S10. D) 2020–2022 survival probability per vaccine doses before COVID-19 and COVID-19 treatment, 1–2 vaccines. AVs, antivirals; MoABs, monoclonal antibodies. ∗No treatment includes patients that did not need to receive treatment mainly due to lack of symptoms. This figure has been replicated with the survival probability ranging from 70% to 100% in Supplementary Fig. S11. E) 2020–2022 survival probability per vaccine doses before COVID-19 and COVID-19 treatment, 3+ vaccines. AVs, antivirals; MoABs, monoclonal antibodies. ∗No treatment includes patients that did not need to receive treatment mainly due to lack of symptoms. This figure has been replicated with the survival probability ranging from 70% to 100% in Supplementary Fig. S12.

Moreover, the study demonstrated a significant increase in the likelihood of surviving up to day 30 after being diagnosed with COVID-19, especially for patients who received their diagnosis later in the pandemic (Fig. 3A). Vaccinated individuals also had considerably higher survival rates when compared to the unvaccinated (Fig. 3B). This increase in survival probability was notable regardless of initial malignancy status (Supplementary Fig. S3A), or setting during their COVID-19 episode at home, in hospital, or ICU (Supplementary Fig. S3B).

The impact of COVID-19 treatment on survival was substantial. For unvaccinated patients, those with the highest likelihood of survival were either those who did not require any COVID-19 treatment or those who received monoclonal antibodies alone or in combination with antiviral medications (Fig. 3C). Among patients who had received 1–2 vaccine doses, highest survival probability was with no treatment required or treatment involving monoclonal antibodies and antivirals alone or in combination (Fig. 3D). Finally, for patients who had received 3 or more vaccinations, various treatment options showed varying degrees of effectiveness, except for corticosteroids alone (Fig. 3E).

## Discussion

This manuscript, encompassing data from 2020 to 2022, aims to shed light on the significant impact of COVID-19 on hematological malignancy patients, with a particular emphasis on mortality, which was specifically pronounced in the year 2020. The study uncovers several critical factors that influence negatively COVID-19 outcomes in this patient population, with age, comorbidities, and malignancy status emerging as key risk factors for mortality. In contrast, the introduction of COVID-19 vaccines and the progression of the pandemic into later phases, leading to improved preventive and targeted treatment options, heightened awareness, earlier treatment initiation, and a broader knowledge base overall, are associated with reduced mortality. Additionally, the study delves into the evolving landscape of hematological malignancies most closely linked to COVID-19 occurrences, revealing dynamic shifts in prevalence that necessitate adaptable treatment strategies to address emerging challenges. These findings underscore the significance of tailored care, ongoing monitoring, and flexibility in managing patients with hematological malignancies in the ever-evolving context of COVID-19.

The evolving landscape of hematological malignancies presents a noteworthy development. While the number of reported COVID-19 cases in non-Hodgkin lymphoma patients remained consistent over time, there was an increase in reported cases among individuals with plasma cell disorders and acute leukemia. Conversely, the number of cases in patients with myelodysplastic syndrome and chronic myeloid malignancies decreased. These shifts underscore the importance of continuously adapting treatment strategies to address new challenges and devising personalized approaches to manage patients with hematological malignancies during a pandemic. Taken together, these data reveal an overall predominance of COVID-19 diagnoses among patients with lymphoid malignancies throughout the years. This could potentially be attributed to the well-documented lower efficacy of anti-SARS-CoV-2 vaccines and their reduced development of immunity in this patient population.^7^^,^^9^^,^^14^

The most encouraging findings center around the effects of COVID-19 vaccination and treatment strategies. During the research period, vaccination emerged as a significant approach in mitigating the severity of COVID-19, even in patients with hematological malignancies.^7^ Although it was not possible to establish complete immunity due to data limitations, the data did indicate a dose-response relationship. Patients who had received a greater number of vaccine doses before being diagnosed with COVID-19 experienced significantly lower mortality rates, affirming the findings from our initial report.^31^ This underscores the importance of vaccination programs and booster doses in offering the utmost protection to this highly vulnerable group. Additionally, early therapeutic strategies, particularly the use of targeted antivirals and monoclonal antibodies, were crucial in enhancing patient outcomes.^35^, ^36^, ^37^ These early treatments not only reduced the severity of the illness but also led to decreased mortality rates.^10^^,^^11^^,^^18^

The study also revealed a notable decline in the severity of COVID-19 cases as time progressed. This trend aligns with the broader context of the pandemic, where advancements in treatment, including the early administration of drugs, and the implementation of prophylaxis and vaccine programs ultimately led to milder cases.^38^ The reduction in COVID-19 mortality across various severity levels, all-cause mortality, and factors contributing to mortality is a testament to the collaborative efforts of the medical community. Patients have reaped the rewards of enhanced treatments and more effective supportive care, as healthcare professionals have become increasingly skilled at early detection and intervention. These trends underscore the adaptability and resilience of the medical community in the midst of a rapidly evolving health crisis. Nonetheless, it is important to remain vigilant, as the severity levels in hematological malignancy patients are still elevated compared to the general population.

One of the most striking findings in the dataset is the increase in survival probability at day 30 following a COVID-19 diagnosis. Patients diagnosed later in the pandemic showed notably higher survival rates. Vaccinated individuals also exhibited significantly improved survival rates. This proves that not only have medical approaches advanced, but patient outcomes have benefited from the accumulation of clinical experience and learning over time. Moreover, the impact of COVID-19-specific treatments on survival should not be underestimated. The study revealed that among unvaccinated patients, those who received monoclonal antibodies alone or in combination with antiviral medications had the highest likelihood of survival, aligning with findings from studies conducted on the general population.^39^ This underscores the importance of tailoring treatment options based on vaccination status and the severity of illness of each patient.

Despite positive trends and progress, the challenge of COVID-19 persists for hematological malignancy patients, particularly those who are in-hospital and critically ill. While the World Health Organization (WHO) has declared the pandemic phase to be over, concerns still loom regarding the virus's tendency to mutate and the potential reduction in the effectiveness of preventive measures. The EPICOVIDEHA registry has demonstrated its value as a valuable tool for surveillance and research in this regard.^2^^,^^7^^,^^8^^,^^10^^,^^11^^,^^13^^,^^19^, ^20^, ^21^^,^^24^, ^25^, ^26^^,^^28^^,^^29^^,^^31^^,^^33^ As the virus continues to evolve, vigilance and adaptability are of paramount importance. In the midst of a constantly shifting landscape, continuous surveillance, research, and adaptation will be crucial to ensure that patients with hematological malignancies receive the highest quality of care. Ongoing monitoring and adjustment of strategies are needed to address the potential impact of viral mutations and any declines in the effectiveness of preventive measures. The EPICOVIDEHA registry stands as a vital tool in this ongoing battle, offering valuable insights into the ever-changing landscape of COVID-19 in hematological malignancy patients.^32^

However, our study has several limitations. Firstly, there is the potential for selection bias due to the use of retrospective data from specific institutions. Being an observational study, the results may be influenced by confounding variables and potential reporting bias stemming from self-reported data. Factors that vary with time and the validation process could also impact the findings, and the study's design makes it difficult to establish causation reliably. Additionally, the absence of viral sequencing and serological data for all patients may limit a comprehensive understanding of patient risk and the natural history of infection. Furthermore, the lack of specific denominators due to the scarcity of cases hinders the ability to provide critical information. Further analyses should also provide information with this regard. Additionally, such analyses would also benefit from reporting the days between the different vaccine doses and the baseline malignancy diagnosis or the existence of previous COVID-19 episodes, as this may impact in the evolution of the infectious episode, specially in the last semesters. Ultimately, the diverse international and multicentric approach adopted in this manuscript may have introduced variability in the findings. This variation could be attributed to differing timelines in the implementation of preventive measures, variations in vaccine types, discrepancies in diagnostic test performance, or even disparities in the availability of treatments for COVID-19. It is inherently challenging to determine the number of SARS-CoV-2-infected patients treated at the participating institutions who went undiagnosed during this timeframe. However, it is plausible that the figures for 2022, particularly in the last six months, are affected by individuals with mild infections who may not have sought medical attention, and physicians might not have actively pursued virologic diagnoses for patients with mild symptoms. Consequently, these cases cannot be incorporated into the report, and it is imperative to recognize this as a limitation. Finally, to enhance the accessibility of our findings for our target audience, primarily hematologists and infectious diseases medical professionals, who may lack advanced statistical proficiency, we have streamlined our analysis to strike a balance between complexity and clarity. As such, we have excluded intricate analyses within our multivariable Cox regression model, recognizing that not all variables maintain proportionality over time or linearity. This omission of comprehensive analysis may result in conservative estimations, particularly impacting variables such as vaccination status as a protective factor and patient stay during the COVID-19 episode as a risk factor. In simpler terms, the results from the multivariable Cox regression model would require adjustments based on the specific duration of exposure. Consequently, the hazard ratio for vaccination may decrease over time with increased vaccine doses (being the vaccines even more protective than what reported here), while it may rise with hospital or ICU admission during the COVID-19 episode (being these admissions even more risky than what reported here). Given these considerations, while urging caution in interpreting our findings, we recommend future analyses to address this limitation. Despite these limitations, the study offers valuable insights into COVID-19 outcomes in patients with hematological malignancies, expanding our knowledge in this field.

In conclusion, the data from the EPICOVIDEHA registry offer a multifaceted view of the COVID-19 pandemic for patients with hematological malignancies. While there have been improvements, the road ahead remains challenging. The findings from this extensive dataset underscore the critical importance of vaccination, prophylactic measures, and vigilant monitoring. As the COVID-19 situation continues to evolve, we must persist in learning, adapting, and advocating for the well-being of patients with hematological malignancies. Through ongoing research and collaboration, we can navigate the ever-changing COVID-19 landscape and provide the best possible care for this vulnerable patient population.

## Contributors

JSG, FM, LP and OAC contributed to study design and study supervision. JSG did the statistical plan and analysis. JSG and OAC interpreted the data and wrote the paper. All the authors recruited, and documented participants, critically read, reviewed, and agreed to publish the manuscript.

## Data sharing statement

The corresponding author can provide the data supporting the findings of this study upon a reasonable request.

## Declaration of interests

**JSG** has received payment or honoraria for lectures, presentations, speakers bureaus, manuscript writing or educational events from Gilead, Menarini, and Pfizer; and has participated on a Data Safety Monitoring Board or Advisory Board for Pfizer, outside of the submitted work.

**FI** has received payment or honoraria for lectures, presentations, speakers bureaus, manuscript writing or educational events from Novartis, AbbVie, Gilead; and has received support for attending meetings and/or travel from Novartis, AbbVie, Gilead, Astellas, Pfizer, Sanofi, BMS, Alexion, Astra-Zeneca, outside of the submitted work.

**MGdS** has received grants or contracts from AstraZeneca, consulting fees from Roche, Janssen Cilag, Gilead, and Abbvie; payment or honoraria for lectures, presentations, speakers bureaus, manuscript writing or educational events from Janssen; support for attending meetings and/or travel from Abbvie, Gilead, and Takeda; and participation on a Data Safety Monitoring Board or Advisory Board for Roche, Janssen Cilag, Lilly, Gilead, Takeda, and Abbvie, outside of the submitted work.

**ALG** has received consulting fees from AstraZeneca; payment or honoraria for lectures, presentations, speakers bureaus, manuscript writing or educational events from Roche, Janssen, and Abbvie; and support for attending from meetings and/or travel from Astrazeneca, Janssen, and Beigene, outside of the submitted work.

**CGV** has received grants or contracts from Ministerio de Sanidad y Consumo, Instituto de Salud Carlos III, payment or honoraria for lectures, presentations, speakers bureaus, manuscript writing or educational events from Gilead Science, MSD, Pfizer, Jannsen, Novartis, Basilea, GSK, Shionogi, AbbVie, Advanz Pharma, and a grant support from Gilead Science, Pfizer, GSK, MSD and Pharmamar, outside of the submitted work.

**MM** has received payment or honoraria for lectures, presentations, speakers bureaus, manuscript writing or educational events from Pfizer, GILEAD, MSD, and ViiV Healthcare; and support for attending meetings and/or travel from Pfizer, Pharmamar, Tillotts Pharma, outside of the submitted work.

**SKG** has received grants or contracts from Else Kröner-Fresenius-Stiftung iPRIME Scholarship (2021_EKPK.10), UKE, Hamburg, outside of the submitted work.

**AV** has received consulting fees from MSD and Takeda; Payment or honoraria for lectures, presentations, speakers bureaus, manuscript writing or educational events from Gilead Pharma; and support for attending meetings and/or travel from Tillots, outside of the submitted work.

**TFA** has received a pre-doctoral grant supported by the Ministerio de Sanidad y Consumo, Instituto de Salud Carlos III [RH RH042953], CM23/00277, outside of the submitted work.

**OAC** has received grants or contracts from BMBF, Cidara, EU-DG RTD (101037867), F2G, Gilead, MedPace, MSD, Mundipharma, Octapharma, Pfizer, Scynexis; consulting fees from Abbvie, AiCuris, Biocon, Cidara, Gilead, IQVIA, Janssen, Matinas, MedPace, Menarini, Moderna, Molecular Partners, MSG-ERC, Noxxon, Octapharm, Pfizer, PSI, Scynexis, Seres; payment or honoraria for lectures, presentations, speakers bureaus, manuscript writing or educational events from Abbott, Abbvie, Al-Jazeera Pharmaceuticals/Hikma, Gilead, Grupo Biotoscana/United Medical/Knight, MedScape, MedUpdate, Merck/MSD, Noscendo, Pfizer, Shionogi, streamedup!; Payment for expert testimony from Cidara; a German patent (“Geschlossene Inkubationssysteme mit verbessertem Atemwegszugang für Untersuchungsvorrichtungen”, DE 10 2021 113 007.7), filed by the University of Cologne and listing Oliver A. Cornely as one of three inventors; Participation on a Data Safety Monitoring Board or Advisory Board from Boston Strategic Partners, Cidara, IQVIA, Janssen, MedPace, PSI, Pulmocide, Shionogi, The Prime Meridian Group; Stock or stock options from CoRe Consulting, EasyRadiology; and Other financial or non-financial interests from Wiley, outside of the submitted work.

**JM** has received consulting fees from Takeda, F2G, and Mundipharma; payment or honoraria for lectures, presentations, speakers bureaus, manuscript writing or educational events from Takeda, F2G, and Mundipharma; and participation on a data safety monitoring board or advisory board from Takeda and Mundipharma, outside of the submitted work.

**JB** has received payment or honoraria for lectures, presentations, speakers bureaus, manuscript writing or educational events from Janseen, Takeda and Pfizer and support for attending meetings and/or travel from Janssen and Pfizer, outside of the submitted work.

**ASO** has received payment or honoraria for lectures, presentations, speakers bureaus, manuscript writing or educational events from Pfizer, Gilead, MSD, and BioMeriuex, outside of the submitted work.

**CDR** has received grants or contracts from Asociación Española Contra el Cáncer: Code Grant: CLJUN18010DERA (01/10/18–30/11/22), outside of the submitted work.

**NK** has received propatient research grant: Third Party Donor Registry for personalized antiviral T-Cell immunotherapeutics, no. pp 20–34; SNSF: Epstein–Barr virus-specific T memory stem cell therapy, Projektförderung (Abt. I-III), no. 204944, SNSF: NCCR AntiResist, no. 180541; consulting fees from MSD Sharp & Dome, Pfizer, Gilead Sciences, and Takeda; patents planes, issued or pending for corss protective epitopes of *Aspergillus fumigatus* and *Candida albicans*; participation on a data safety mornitoing board or advisory board or Idorsia and Pulmocide; and Leadership or fiduciary role in other board, society, committee or advocacy groups, paid or unpaid for Fungal Infection Network of Switzerland (FUNGINOS), outside of the submitted work.

**RDB** has been conference speaker to Novartis, Kite/Gilead, Pfizer, Abbie, and Incyte; has received travel accommodation from Kite/Gilead; and has participated in Scientific advisory board for Novartis, Kite/Gilead, Janssen, and BMS, outside of the submitted work.

**JAHR** has received grants or contracts from BMS/Celgene, Janssen, Sanofi, and GSK; Consulting fees from Janssen, Roche, Abbvie, Gilead, BMS/Celgene, Amgen, Takeda, Rovi, AstraZeneca, Sandoz Novartis, Celltrion, EusaPharm, Sanofi, Beigene, and Lilly; and payment or honoraria for lectures, presentations, speakers bureaus, manuscript writing or educational events from Janssen, Roche, Abbvie, Gilead, BMS/Celgene, Amgen, Takeda, AstraZeneca, Beigene, Lilly, and GSK, outside of the submitted work.

**LD** has received payment or honoraria for lectures, presentations, speakers bureaus, manuscript writing or educational events from Pfizer; and support for attending meetings and/or travel from Pfizer, outside of the submitted work.

**PK** has received grants from German Federal Ministry of Research and Education (BMBF) B-FAST (Bundesweites Forschungsnetz Angewandte Surveillance und Testung) and NAPKON (Nationales Pandemie Kohorten Netz, German National Pandemic Cohort Network) of the Network University Medicine (NUM) and the State of North Rhine-Westphalia; consulting fees from Ambu GmbH, Gilead Sciences, Mundipharma Resarch Limited, Noxxon N.V., Pfizer Pharma; payment or honoraria for lectures, presentations, speakers bureaus, manuscript writing or educational events from Akademie für Infektionsmedizin e.V., Ambu GmbH, Astellas Pharma, BioRad Laboratories Inc., European Confederation of Medical Mycology, Gilead Sciences, GPR Academy Ruesselsheim, HELIOS Kliniken GmbH, Jazz Pharmaceuticals Germany GmbH, medupdate GmbH, MedMedia GmbH, MSD Sharp & Dohme GmbH, Pfizer Pharma GmbH, Scilink Comunicación Científica SC and University Hospital, LMU Munich; A German patent application (“Geschlossene Intubationssysteme mit verbessertem Atemwegszugang für Untersuchungsvorrichtungen”, official file number DE 10 2021 113 007.7) has been filed by the University of Cologne; Participation on a Data Safety Monitoring Board or Advisory Board from Ambu GmbH, Gilead Sciences, Pfizer Pharma, Mundipharma Resarch Limited, Noxxon N.V.; and Other financial or non-financial interests from Elsevier, Wiley, outside of the submitted work.

**PJ** has received Payment or honoraria for lectures, presentations, speakers bureaus, manuscript writing or educational events from GSK; Payment for expert testimony from Takeda; Support for attending meetings and/or travel from AstraZeneca, Novartis; and Participation on a Data Safety Monitoring Board or Advisory Board Takeda, BMS, outside of the submitted work.

All authors had full access to the data and had final responsibility for the decision to submit for publication.

## Appendix A Supplementary data

Supplementary Fig. S12

R3. Collaborators
