# Supplementary material for: Decoding the historical tale: COVID-19 impact on haematological malignancy patients—EPICOVIDEHA insights from 2020 to 2022
Source: eClinicalMedicine. 2024 Mar 18;71:102553. doi: 10.1016/j.eclinm.2024.102553 (PMC10963230; doi:10.1016/j.eclinm.2024.102553)

**DECODING THE HISTORICAL TALE: COVID-19 IMPACT ON HAEMATOLOGICAL MALIGNANCY PATIENTS – EPICOVIDEHA INSIGHTS FROM 2020 TO 2022**

**SUPPLEMENTARY TABLES**

**Supplementary table 1.** Characteristics of EPICOVIDEHA patients: January-June 2020

|  | **January-June 2020** | | | | | |
| --- | --- | --- | --- | --- | --- | --- |
|  | ***Total*** | | ***Alive*** | | ***Dead*** | |
|  | **n** | **%** | **n** | **%** | **n** | **%** |
| **Sex** |  |  |  |  |  |  |
| Female | 617 | 37.0 | 362 | 58.7 | 255 | 41.3 |
| Male | 1052 | 63.0 | 608 | 57.8 | 444 | 42.2 |
| **Age** | 68 (57-77) [18-97] | | 64 (54-73) [18-97] | | 73 (63-81) [20-94] | |
| 18-25 years old | 31 | 1.9 | 19 | 61.3 | 12 | 38.7 |
| 26-50 years old | 225 | 13.5 | 174 | 77.3 | 51 | 22.7 |
| 51-69 years old | 636 | 38.1 | 434 | 68.2 | 202 | 31.8 |
| >69 years old | 777 | 46.6 | 343 | 44.1 | 434 | 55.9 |
| **Comorbidities at COVID-19 onset** |  |  |  |  |  |  |
| 0-1 comorbidities | 1037 | 62.1 | 660 | 63.6 | 377 | 36.4 |
| 2+ comorbidities | 632 | 37.9 | 310 | 49.1 | 322 | 50.9 |
| **Baseline hematological malignancy** |  |  |  |  |  |  |
| Hodgkin lymphoma | 50 | 3.0 | 38 | 3.9 | 12 | 1.7 |
| Chronic lymphoid leukemia | 231 | 13.8 | 159 | 16.4 | 72 | 10.3 |
| Acute leukemia | 205 | 12.3 | 83 | 8.6 | 122 | 17.5 |
| Non-Hodgkin lymphoma | 555 | 33.3 | 323 | 33.3 | 232 | 33.2 |
| Chronic myeloid malignancies | 198 | 11.9 | 134 | 13.8 | 64 | 9.2 |
| Plasma cell disorders | 275 | 16.5 | 164 | 16.9 | 111 | 15.9 |
| Myelodysplastic syndrome | 141 | 8.4 | 59 | 6.1 | 82 | 11.7 |
| Hairy cell leukemia | 14 | 0.8 | 10 | 1.0 | 4 | 0.6 |
| **Baseline hematological malignancy status at COVID-19 onset** |  |  |  |  |  |  |
| Controlled malignancy | 738 | 44.2 | 518 | 70.2 | 220 | 29.8 |
| Stable malignancy | 298 | 17.9 | 175 | 58.7 | 123 | 41.3 |
| Active malignancy | 553 | 33.1 | 246 | 44.5 | 307 | 55.5 |
| **SARS-CoV-2 vaccination at COVID-19 onset** |  |  |  |  |  |  |
| 0 doses | 1669 | 100.0 | 970 | 58.1 | 699 | 41.9 |
| Not vaccinated | 1669 | 100.0 | 970 | 58.1 | 699 | 41.9 |
| 1+2 doses | 0 | 0.0 | 0 | 0.0% | 0 | 0.0% |
| One dose | 0 | 0.0 | 0 | 0.0% | 0 | 0.0% |
| Two doses | 0 | 0.0 | 0 | 0.0% | 0 | 0.0% |
| 3+ doses | 0 | 0.0 | 0 | 0.0% | 0 | 0.0% |
| Three doses | 0 | 0.0 | 0 | 0.0% | 0 | 0.0% |
| Four doses | 0 | 0.0 | 0 | 0.0% | 0 | 0.0% |
| Five doses | 0 | 0.0 | 0 | 0.0% | 0 | 0.0% |
| **Stay during COVID-19** |  |  |  |  |  |  |
| Home | 260 | 15.6 | 223 | 85.8 | 37 | 14.2 |
| Hospital no ICU | 1090 | 65.3 | 621 | 57.0 | 469 | 43.0 |
| Hospital ICU | 319 | 19.1 | 126 | 39.5 | 193 | 60.5 |
| **COVID-19 treatment** |  |  |  |  |  |  |
| No treatment | 122 | 7.3 | 72 | 59.0 | 50 | 41.0 |
| Antivirals ± plasma ± corticosteroids | 19 | 1.1 | 10 | 52.6 | 9 | 47.4 |
| Antivirals + monoclonal antibodies ± plasma ± corticosteroids | 0 | 0.0 | 0 | 0.0 | 0 | 0.0 |
| Monoclonal antibodies ± plasma ± corticosteroids | 1 | 0.1 | 1 | 100.0 | 0 | 0.0 |
| Corticosteroids | 83 | 5.0 | 45 | 54.2 | 38 | 45.8 |
| Plasma ± corticosteroids | 8 | 0.5 | 4 | 50.0 | 4 | 50.0 |
| Unknown | 1436 | 86.0 | 838 | 58.4 | 598 | 41.6 |
| **Day 30 outcome** |  |  |  |  |  |  |
| Alive | 1191 | 71.4 |  |  |  |  |
| Dead | 478 | 28.6 |  |  |  |  |
| *Reason for mortality* |  |  |  |  |  |  |
| COVID-19 | 336 | 70.3 |  |  | 336 | 70.3 |
| COVID-19 + hematological malignancy | 84 | 17.6 |  |  | 84 | 17.6 |
| Hematological malignancies +/- other reasons | 58 | 12.1 |  |  | 58 | 12.1 |

COVID-19, coronavirus disease 2019; ICU, intensive care unit; SARS-CoV-2, severe acute respiratory syndrome coronavirus 2

** No treatment includes patients that did not need to receive treatment mainly due to lack of symptoms.*

**Supplementary table 2.** Characteristics of EPICOVIDEHA patients: July-December 2020

|  | **July-December 2020** | | | | | |
| --- | --- | --- | --- | --- | --- | --- |
|  | ***Total*** | | ***Alive*** | | ***Dead*** | |
|  | **n** | **%** | **n** | **%** | **n** | **%** |
| **Sex** |  |  |  |  |  |  |
| Female | 1032 | 44.0 | 777 | 75.3 | 255 | 24.7 |
| Male | 1315 | 56.0 | 932 | 70.9 | 383 | 29.1 |
| **Age** | 64 (52-73) [18-97] | | 61 (50-72) [18-95] | | 69 (60-77) [18-97] | |
| 18-25 years old | 73 | 3.1 | 62 | 84.9 | 11 | 15.1 |
| 26-50 years old | 450 | 19.2 | 390 | 86.7 | 60 | 13.3 |
| 51-69 years old | 995 | 42.4 | 745 | 74.9 | 250 | 25.1 |
| >69 years old | 829 | 35.3 | 512 | 61.8 | 317 | 38.2 |
| **Comorbidities at COVID-19 onset** |  |  |  |  |  |  |
| 0-1 comorbidities | 1707 | 72.7 | 1300 | 76.2 | 407 | 23.8 |
| 2+ comorbidities | 640 | 27.3 | 409 | 63.9 | 231 | 36.1 |
| **Baseline hematological malignancy** |  |  |  |  |  |  |
| Hodgkin lymphoma | 110 | 4.7 | 104 | 6.1 | 6 | 0.9 |
| Chronic lymphoid leukemia | 279 | 11.9 | 200 | 11.7 | 79 | 12.4 |
| Acute leukemia | 454 | 19.3 | 290 | 17.0 | 164 | 25.7 |
| Non-Hodgkin lymphoma | 624 | 26.6 | 447 | 26.2 | 177 | 27.7 |
| Chronic myeloid malignancies | 276 | 11.8 | 240 | 14.0 | 36 | 5.6 |
| Plasma cell disorders | 435 | 18.5 | 314 | 18.4 | 121 | 19.0 |
| Myelodysplastic syndrome | 149 | 6.3 | 100 | 5.9 | 49 | 7.7 |
| Hairy cell leukemia | 20 | 0.9 | 14 | 0.8 | 6 | 0.9 |
| **Baseline hematological malignancy status at COVID-19 onset** |  |  |  |  |  |  |
| Controlled malignancy | 1148 | 48.9 | 941 | 82.0 | 207 | 18.0 |
| Stable malignancy | 350 | 14.9 | 279 | 79.7 | 71 | 20.3 |
| Active malignancy | 791 | 33.7 | 458 | 57.9 | 333 | 42.1 |
| **SARS-CoV-2 vaccination at COVID-19 onset** |  |  |  |  |  |  |
| 0 doses | 2347 | 100.0 | 1709 | 72.8 | 638 | 27.2 |
| Not vaccinated | 2347 | 100.0 | 1709 | 72.8 | 638 | 27.2 |
| 1+2 doses | 0 | 0.0 | 0 | 0.0% | 0 | 0.0% |
| One dose | 0 | 0.0 | 0 | 0.0% | 0 | 0.0% |
| Two doses | 0 | 0.0 | 0 | 0.0% | 0 | 0.0% |
| 3+ doses | 0 | 0.0 | 0 | 0.0% | 0 | 0.0% |
| Three doses | 0 | 0.0 | 0 | 0.0% | 0 | 0.0% |
| Four doses | 0 | 0.0 | 0 | 0.0% | 0 | 0.0% |
| Five doses | 0 | 0.0 | 0 | 0.0% | 0 | 0.0% |
| **Stay during COVID-19** |  |  |  |  |  |  |
| Home | 885 | 37.7 | 849 | 95.9 | 36 | 4.1 |
| Hospital no ICU | 1061 | 45.2 | 737 | 69.5 | 324 | 30.5 |
| Hospital ICU | 401 | 17.1 | 123 | 30.7 | 278 | 69.3 |
| **COVID-19 treatment** |  |  |  |  |  |  |
| No treatment | 178 | 7.6 | 154 | 86.5 | 24 | 13.5 |
| Antivirals ± plasma ± corticosteroids | 99 | 4.2 | 45 | 45.5 | 54 | 54.5 |
| Antivirals + monoclonal antibodies ± plasma ± corticosteroids | 6 | 0.3 | 4 | 66.7 | 2 | 33.3 |
| Monoclonal antibodies ± plasma ± corticosteroids | 7 | 0.3 | 6 | 85.7 | 1 | 14.3 |
| Corticosteroids | 149 | 6.3 | 77 | 51.7 | 72 | 48.3 |
| Plasma ± corticosteroids | 30 | 1.3 | 20 | 66.7 | 10 | 33.3 |
| Unknown | 1878 | 80.0 | 1403 | 74.7 | 475 | 25.3 |
| **Day 30 outcome** |  |  |  |  |  |  |
| Alive | 1927 | 82.1 |  |  |  |  |
| Dead | 420 | 17.9 |  |  |  |  |
| *Reason for mortality* |  |  |  |  |  |  |
| COVID-19 | 276 | 65.7 |  |  | 276 | 65.7 |
| COVID-19 + hematological malignancy | 110 | 26.2 |  |  | 110 | 26.2 |
| Hematological malignancies +/- other reasons | 34 | 8.1 |  |  | 34 | 8.1 |

COVID-19, coronavirus disease 2019; ICU, intensive care unit; SARS-CoV-2, severe acute respiratory syndrome coronavirus 2

** No treatment includes patients that did not need to receive treatment mainly due to lack of symptoms.*

**Supplementary table 3.** Characteristics of EPICOVIDEHA patients: January-June 2021

|  | **January-June 2021** | | | | | |
| --- | --- | --- | --- | --- | --- | --- |
|  | ***Total*** | | ***Alive*** | | ***Dead*** | |
|  | **n** | **%** | **n** | **%** | **n** | **%** |
| **Sex** |  |  |  |  |  |  |
| Female | 325 | 38.6 | 238 | 73.2 | 87 | 26.8 |
| Male | 517 | 61.4 | 363 | 70.2 | 154 | 29.8 |
| **Age** | 64 (51-73) [18-106] | | 62 (49-72) [18-94] | | 68 (57-78) [18-106] | |
| 18-25 years old | 38 | 4.5 | 29 | 76.3 | 9 | 23.7 |
| 26-50 years old | 167 | 19.8 | 133 | 79.6 | 34 | 20.4 |
| 51-69 years old | 345 | 41.0 | 254 | 73.6 | 91 | 26.4 |
| >69 years old | 292 | 34.7 | 185 | 63.4 | 107 | 36.6 |
| **Comorbidities at COVID-19 onset** |  |  |  |  |  |  |
| 0-1 comorbidities | 611 | 72.6 | 455 | 74.5 | 156 | 25.5 |
| 2+ comorbidities | 231 | 27.4 | 146 | 63.2 | 85 | 36.8 |
| **Baseline hematological malignancy** |  |  |  |  |  |  |
| Hodgkin lymphoma | 41 | 4.9 | 36 | 6.0 | 5 | 2.1 |
| Chronic lymphoid leukemia | 143 | 17.0 | 100 | 16.6 | 43 | 17.8 |
| Acute leukemia | 160 | 19.0 | 94 | 15.6 | 66 | 27.4 |
| Non-Hodgkin lymphoma | 249 | 29.6 | 177 | 29.5 | 72 | 29.9 |
| Chronic myeloid malignancies | 79 | 9.4 | 70 | 11.6 | 9 | 3.7 |
| Plasma cell disorders | 111 | 13.2 | 83 | 13.8 | 28 | 11.6 |
| Myelodysplastic syndrome | 51 | 6.1 | 34 | 5.7 | 17 | 7.1 |
| Hairy cell leukemia | 8 | 1.0 | 7 | 1.2 | 1 | 0.4 |
| **Baseline hematological malignancy status at COVID-19 onset** |  |  |  |  |  |  |
| Controlled malignancy | 325 | 38.6 | 253 | 77.8 | 72 | 22.2 |
| Stable malignancy | 211 | 25.1 | 157 | 74.4 | 54 | 25.6 |
| Active malignancy | 269 | 31.9 | 171 | 63.6 | 98 | 36.4 |
| **SARS-CoV-2 vaccination at COVID-19 onset** |  |  |  |  |  |  |
| 0 doses | 667 | 79.2 | 462 | 69.3 | 205 | 30.7 |
| Not vaccinated | 667 | 79.2 | 462 | 69.3 | 205 | 30.7 |
| 1+2 doses | 175 | 20.8 | 139 | 79.4 | 36 | 20.6 |
| One dose | 91 | 10.8 | 75 | 82.4 | 16 | 17.6 |
| Two doses | 84 | 10.0 | 64 | 76.2 | 20 | 23.8 |
| 3+ doses | 0 | 0.0 | 0 | 0.0% | 0 | 0.0% |
| Three doses | 0 | 0.0 | 0 | 0.0% | 0 | 0.0% |
| Four doses | 0 | 0.0 | 0 | 0.0% | 0 | 0.0% |
| Five doses | 0 | 0.0 | 0 | 0.0% | 0 | 0.0% |
| **Stay during COVID-19** |  |  |  |  |  |  |
| Home | 206 | 24.5 | 199 | 96.6 | 7 | 3.4 |
| Hospital no ICU | 456 | 54.2 | 343 | 75.2 | 113 | 24.8 |
| Hospital ICU | 180 | 21.4 | 59 | 32.8 | 121 | 67.2 |
| **COVID-19 treatment** |  |  |  |  |  |  |
| No treatment | 158 | 18.8 | 139 | 88.0 | 19 | 12.0 |
| Antivirals ± plasma ± corticosteroids | 135 | 16.0 | 89 | 65.9 | 46 | 34.1 |
| Antivirals + monoclonal antibodies ± plasma ± corticosteroids | 23 | 2.7 | 20 | 87.0 | 3 | 13.0 |
| Monoclonal antibodies ± plasma ± corticosteroids | 43 | 5.1 | 40 | 93.0 | 3 | 7.0 |
| Corticosteroids | 237 | 28.1 | 132 | 55.7 | 105 | 44.3 |
| Plasma ± corticosteroids | 37 | 4.4 | 22 | 59.5 | 15 | 40.5 |
| Unknown | 209 | 24.8 | 159 | 76.1 | 50 | 23.9 |
| **Day 30 outcome** |  |  |  |  |  |  |
| Alive | 683 | 81.1 |  |  |  |  |
| Dead | 159 | 18.9 |  |  |  |  |
| *Reason for mortality* |  |  |  |  |  |  |
| COVID-19 | 90 | 56.6 |  |  | 90 | 56.6 |
| COVID-19 + hematological malignancy | 54 | 34.0 |  |  | 54 | 34.0 |
| Hematological malignancies +/- other reasons | 15 | 9.4 |  |  | 15 | 9.4 |

COVID-19, coronavirus disease 2019; ICU, intensive care unit; SARS-CoV-2, severe acute respiratory syndrome coronavirus 2

** No treatment includes patients that did not need to receive treatment mainly due to lack of symptoms.*

**Supplementary table 4.** Characteristics of EPICOVIDEHA patients: July-December 2021

|  | **July-December 2021** | | | | | |
| --- | --- | --- | --- | --- | --- | --- |
|  | ***Total*** | | ***Alive*** | | ***Dead*** | |
|  | **n** | **%** | **n** | **%** | **n** | **%** |
| **Sex** |  |  |  |  |  |  |
| Female | 445 | 42.9 | 356 | 80.0 | 89 | 20.0 |
| Male | 593 | 57.1 | 475 | 80.1 | 118 | 19.9 |
| **Age** | 65 (54-75) [18-95] | | 63 (51-74) [18-95] | | 71 (61-78) [27-95] | |
| 18-25 years old | 38 | 3.7 | 38 | 100.0 | 0 | 0.0 |
| 26-50 years old | 178 | 17.1 | 161 | 90.4 | 17 | 9.6 |
| 51-69 years old | 423 | 40.8 | 347 | 82.0 | 76 | 18.0 |
| >69 years old | 399 | 38.4 | 285 | 71.4 | 114 | 28.6 |
| **Comorbidities at COVID-19 onset** |  |  |  |  |  |  |
| 0-1 comorbidities | 756 | 72.8 | 629 | 83.2 | 127 | 16.8 |
| 2+ comorbidities | 282 | 27.2 | 202 | 71.6 | 80 | 28.4 |
| **Baseline hematological malignancy** |  |  |  |  |  |  |
| Hodgkin lymphoma | 39 | 3.8 | 34 | 4.1 | 5 | 2.4 |
| Chronic lymphoid leukemia | 149 | 14.4 | 113 | 13.6 | 36 | 17.4 |
| Acute leukemia | 139 | 13.4 | 108 | 13.0 | 31 | 15.0 |
| Non-Hodgkin lymphoma | 374 | 36.0 | 297 | 35.7 | 77 | 37.2 |
| Chronic myeloid malignancies | 100 | 9.6 | 89 | 10.7 | 11 | 5.3 |
| Plasma cell disorders | 178 | 17.1 | 146 | 17.6 | 32 | 15.5 |
| Myelodysplastic syndrome | 51 | 4.9 | 37 | 4.5 | 14 | 6.8 |
| Hairy cell leukemia | 8 | 0.8 | 7 | 0.8 | 1 | 0.5 |
| **Baseline hematological malignancy status at COVID-19 onset** |  |  |  |  |  |  |
| Controlled malignancy | 509 | 49.0 | 432 | 84.9 | 77 | 15.1 |
| Stable malignancy | 233 | 22.4 | 191 | 82.0 | 42 | 18.0 |
| Active malignancy | 246 | 23.7 | 172 | 69.9 | 74 | 30.1 |
| **SARS-CoV-2 vaccination at COVID-19 onset** |  |  |  |  |  |  |
| 0 doses | 247 | 23.8 | 179 | 72.5 | 68 | 27.5 |
| Not vaccinated | 247 | 23.8 | 179 | 72.5 | 68 | 27.5 |
| 1+2 doses | 563 | 54.2 | 457 | 81.2 | 106 | 18.8 |
| One dose | 46 | 4.4 | 38 | 82.6 | 8 | 17.4 |
| Two doses | 517 | 49.8 | 419 | 81.0 | 98 | 19.0 |
| 3+ doses | 228 | 22.0 | 195 | 85.5 | 33 | 14.5 |
| Three doses | 228 | 22.0 | 195 | 85.5 | 33 | 14.5 |
| Four doses | 0 | 0.0 | 0 | 0.0% | 0 | 0.0% |
| Five doses | 0 | 0.0 | 0 | 0.0% | 0 | 0.0% |
| **Stay during COVID-19** |  |  |  |  |  |  |
| Home | 375 | 36.1 | 364 | 97.1 | 11 | 2.9 |
| Hospital no ICU | 500 | 48.2 | 405 | 81.0 | 95 | 19.0 |
| Hospital ICU | 163 | 15.7 | 62 | 38.0 | 101 | 62.0 |
| **COVID-19 treatment** |  |  |  |  |  |  |
| No treatment | 249 | 24.0 | 219 | 88.0 | 30 | 12.0 |
| Antivirals ± plasma ± corticosteroids | 151 | 14.5 | 113 | 74.8 | 38 | 25.2 |
| Antivirals + monoclonal antibodies ± plasma ± corticosteroids | 65 | 6.3 | 50 | 76.9 | 15 | 23.1 |
| Monoclonal antibodies ± plasma ± corticosteroids | 225 | 21.7 | 206 | 91.6 | 19 | 8.4 |
| Corticosteroids | 220 | 21.2 | 134 | 60.9 | 86 | 39.1 |
| Plasma ± corticosteroids | 17 | 1.6 | 11 | 64.7 | 6 | 35.3 |
| Unknown | 111 | 10.7 | 98 | 88.3 | 13 | 11.7 |
| **Day 30 outcome** |  |  |  |  |  |  |
| Alive | 900 | 86.7 |  |  |  |  |
| Dead | 138 | 13.3 |  |  |  |  |
| *Reason for mortality* |  |  |  |  |  |  |
| COVID-19 | 93 | 67.4 |  |  | 93 | 67.4 |
| COVID-19 + hematological malignancy | 40 | 29.0 |  |  | 40 | 29.0 |
| Hematological malignancies +/- other reasons | 5 | 3.6 |  |  | 5 | 3.6 |

COVID-19, coronavirus disease 2019; ICU, intensive care unit; SARS-CoV-2, severe acute respiratory syndrome coronavirus 2

** No treatment includes patients that did not need to receive treatment mainly due to lack of symptoms.*

**Supplementary table 5.** Characteristics of EPICOVIDEHA patients: January-June 2022

|  | **January-June 2022** | | | | | |
| --- | --- | --- | --- | --- | --- | --- |
|  | ***Total*** | | ***Alive*** | | ***Dead*** | |
|  | **n** | **%** | **n** | **%** | **n** | **%** |
| **Sex** |  |  |  |  |  |  |
| Female | 934 | 42.7 | 816 | 87.4 | 118 | 12.6 |
| Male | 1252 | 57.3 | 1063 | 84.9 | 189 | 15.1 |
| **Age** | 66 (53-75) [18-97] | | 64 (52-74) [18-97] | | 71 (59-78) [18-95] | |
| 18-25 years old | 66 | 3.0 | 59 | 89.4 | 7 | 10.6 |
| 26-50 years old | 398 | 18.2 | 367 | 92.2 | 31 | 7.8 |
| 51-69 years old | 847 | 38.7 | 757 | 89.4 | 90 | 10.6 |
| >69 years old | 875 | 40.0 | 696 | 79.5 | 179 | 20.5 |
| **Comorbidities at COVID-19 onset** |  |  |  |  |  |  |
| 0-1 comorbidities | 1602 | 73.3 | 1430 | 89.3 | 172 | 10.7 |
| 2+ comorbidities | 584 | 26.7 | 449 | 76.9 | 135 | 23.1 |
| **Baseline hematological malignancy** |  |  |  |  |  |  |
| Hodgkin lymphoma | 98 | 4.5 | 89 | 4.7 | 9 | 2.9 |
| Chronic lymphoid leukemia | 281 | 12.9 | 241 | 12.8 | 40 | 13.0 |
| Acute leukemia | 387 | 17.7 | 316 | 16.8 | 71 | 23.1 |
| Non-Hodgkin lymphoma | 717 | 32.8 | 616 | 32.8 | 101 | 32.9 |
| Chronic myeloid malignancies | 166 | 7.6 | 152 | 8.1 | 14 | 4.6 |
| Plasma cell disorders | 395 | 18.1 | 347 | 18.5 | 48 | 15.6 |
| Myelodysplastic syndrome | 135 | 6.2 | 112 | 6.0 | 23 | 7.5 |
| Hairy cell leukemia | 7 | 0.3 | 6 | 0.3 | 1 | 0.3 |
| **Baseline hematological malignancy status at COVID-19 onset** |  |  |  |  |  |  |
| Controlled malignancy | 1077 | 49.3 | 999 | 92.8 | 78 | 7.2 |
| Stable malignancy | 455 | 20.8 | 403 | 88.6 | 52 | 11.4 |
| Active malignancy | 597 | 27.3 | 434 | 72.7 | 163 | 27.3 |
| **SARS-CoV-2 vaccination at COVID-19 onset** |  |  |  |  |  |  |
| 0 doses | 542 | 24.8 | 408 | 75.3 | 134 | 24.7 |
| Not vaccinated | 542 | 24.8 | 408 | 75.3 | 134 | 24.7 |
| 1+2 doses | 501 | 22.9 | 441 | 88.0 | 60 | 12.0 |
| One dose | 67 | 3.1 | 62 | 92.5 | 5 | 7.5 |
| Two doses | 434 | 19.9 | 379 | 87.3 | 55 | 12.7 |
| 3+ doses | 1143 | 52.3 | 1030 | 90.1 | 113 | 9.9 |
| Three doses | 1015 | 46.4 | 911 | 89.8 | 104 | 10.2 |
| Four doses | 127 | 5.8 | 119 | 93.7 | 8 | 6.3 |
| Five doses | 1 | 0.0 | 0 | 0.0 | 1 | 100.0 |
| **Stay during COVID-19** |  |  |  |  |  |  |
| Home | 998 | 45.7 | 980 | 98.2 | 18 | 1.8 |
| Hospital no ICU | 998 | 45.7 | 815 | 81.7 | 183 | 18.3 |
| Hospital ICU | 187 | 8.6 | 81 | 43.3 | 106 | 56.7 |
| **COVID-19 treatment** |  |  |  |  |  |  |
| No treatment | 558 | 25.5 | 530 | 95.0 | 28 | 5.0 |
| Antivirals ± plasma ± corticosteroids | 495 | 22.6 | 417 | 84.2 | 78 | 15.8 |
| Antivirals + monoclonal antibodies ± plasma ± corticosteroids | 192 | 8.8 | 158 | 82.3 | 34 | 17.7 |
| Monoclonal antibodies ± plasma ± corticosteroids | 356 | 16.3 | 318 | 89.3 | 38 | 10.7 |
| Corticosteroids | 277 | 12.7 | 184 | 66.4 | 93 | 33.6 |
| Plasma ± corticosteroids | 29 | 1.3 | 23 | 79.3 | 6 | 20.7 |
| Unknown | 279 | 12.8 | 249 | 89.2 | 30 | 10.8 |
| **Day 30 outcome** |  |  |  |  |  |  |
| Alive | 1992 | 91.1 |  |  |  |  |
| Dead | 194 | 8.9 |  |  |  |  |
| *Reason for mortality* |  |  |  |  |  |  |
| COVID-19 | 98 | 50.5 |  |  | 98 | 50.5 |
| COVID-19 + hematological malignancy | 71 | 36.6 |  |  | 71 | 36.6 |
| Hematological malignancies +/- other reasons | 25 | 12.9 |  |  | 25 | 12.9 |

COVID-19, coronavirus disease 2019; ICU, intensive care unit; SARS-CoV-2, severe acute respiratory syndrome coronavirus 2

** No treatment includes patients that did not need to receive treatment mainly due to lack of symptoms.*

**Supplementary table 6.** Characteristics of EPICOVIDEHA patients: July-December 2022

|  | **July-December 2022** | | | | | |
| --- | --- | --- | --- | --- | --- | --- |
|  | ***Total*** | | ***Alive*** | | ***Dead*** | |
|  | **n** | **%** | **n** | **%** | **n** | **%** |
| **Sex** |  |  |  |  |  |  |
| Female | 295 | 43.1 | 272 | 92.2 | 23 | 7.8 |
| Male | 390 | 56.9 | 361 | 92.6 | 29 | 7.4 |
| **Age** | 67 (56-75) [19-95] | | 66 (55-75) [19-93] | | 72 (62-79) [33-95] | |
| 18-25 years old | 22 | 3.2 | 22 | 100.0 | 0 | 0.0 |
| 26-50 years old | 106 | 15.5 | 101 | 95.3 | 5 | 4.7 |
| 51-69 years old | 271 | 39.6 | 256 | 94.5 | 15 | 5.5 |
| >69 years old | 286 | 41.8 | 254 | 88.8 | 32 | 11.2 |
| **Comorbidities at COVID-19 onset** |  |  |  |  |  |  |
| 0-1 comorbidities | 492 | 71.8 | 465 | 94.5 | 27 | 5.5 |
| 2+ comorbidities | 193 | 28.2 | 168 | 87.0 | 25 | 13.0 |
| **Baseline hematological malignancy** |  |  |  |  |  |  |
| Hodgkin lymphoma | 16 | 2.3 | 15 | 2.4 | 1 | 1.9 |
| Chronic lymphoid leukemia | 80 | 11.7 | 76 | 12.0 | 4 | 7.7 |
| Acute leukemia | 130 | 19.0 | 119 | 18.8 | 11 | 21.2 |
| Non-Hodgkin lymphoma | 212 | 30.9 | 192 | 30.3 | 20 | 38.5 |
| Chronic myeloid malignancies | 48 | 7.0 | 46 | 7.3 | 2 | 3.8 |
| Plasma cell disorders | 152 | 22.2 | 142 | 22.4 | 10 | 19.2 |
| Myelodysplastic syndrome | 45 | 6.6 | 41 | 6.5 | 4 | 7.7 |
| Hairy cell leukemia | 2 | 0.3 | 2 | 0.3 | 0 | 0.0 |
| **Baseline hematological malignancy status at COVID-19 onset** |  |  |  |  |  |  |
| Controlled malignancy | 334 | 48.8 | 317 | 94.9 | 17 | 5.1 |
| Stable malignancy | 141 | 20.6 | 133 | 94.3 | 8 | 5.7 |
| Active malignancy | 190 | 27.7 | 164 | 86.3 | 26 | 13.7 |
| **SARS-CoV-2 vaccination at COVID-19 onset** |  |  |  |  |  |  |
| 0 doses | 186 | 27.2 | 164 | 88.2 | 22 | 11.8 |
| Not vaccinated | 186 | 27.2 | 164 | 88.2 | 22 | 11.8 |
| 1+2 doses | 89 | 13.0 | 85 | 95.5 | 4 | 4.5 |
| One dose | 10 | 1.5 | 9 | 90.0 | 1 | 10.0 |
| Two doses | 79 | 11.5 | 76 | 96.2 | 3 | 3.8 |
| 3+ doses | 410 | 59.9 | 384 | 93.7 | 26 | 6.3 |
| Three doses | 241 | 35.2 | 225 | 93.4 | 16 | 6.6 |
| Four doses | 147 | 21.5 | 137 | 93.2 | 10 | 6.8 |
| Five doses | 22 | 3.2 | 22 | 100.0 | 0 | 0.0 |
| **Stay during COVID-19** |  |  |  |  |  |  |
| Home | 354 | 51.7 | 350 | 98.9 | 4 | 1.1 |
| Hospital no ICU | 299 | 43.6 | 264 | 88.3 | 35 | 11.7 |
| Hospital ICU | 29 | 4.2 | 16 | 55.2 | 13 | 44.8 |
| **COVID-19 treatment** |  |  |  |  |  |  |
| No treatment | 124 | 18.1 | 122 | 98.4 | 2 | 1.6 |
| Antivirals ± plasma ± corticosteroids | 296 | 43.2 | 271 | 91.6 | 25 | 8.4 |
| Antivirals + monoclonal antibodies ± plasma ± corticosteroids | 62 | 9.1 | 54 | 87.1 | 8 | 12.9 |
| Monoclonal antibodies ± plasma ± corticosteroids | 28 | 4.1 | 24 | 85.7 | 4 | 14.3 |
| Corticosteroids | 81 | 11.8 | 72 | 88.9 | 9 | 11.1 |
| Plasma ± corticosteroids | 13 | 1.9 | 13 | 100.0 | 0 | 0.0 |
| Unknown | 81 | 11.8 | 77 | 95.1 | 4 | 4.9 |
| **Day 30 outcome** |  |  |  |  |  |  |
| Alive | 659 | 96.2 |  |  |  |  |
| Dead | 26 | 3.8 |  |  |  |  |
| *Reason for mortality* |  |  |  |  |  |  |
| COVID-19 | 11 | 42.3 |  |  | 11 | 42.3 |
| COVID-19 + hematological malignancy | 8 | 30.8 |  |  | 8 | 30.8 |
| Hematological malignancies +/- other reasons | 7 | 26.9 |  |  | 7 | 26.9 |

COVID-19, coronavirus disease 2019; ICU, intensive care unit; SARS-CoV-2, severe acute respiratory syndrome coronavirus 2

** No treatment includes patients that did not need to receive treatment mainly due to lack of symptoms.*

**Supplementary table 7.** Factors associated with mortality in EPICOVIDEHA 2020 – 2022 patients. Sensitivy analysis with missing values in “Days from COVID-19 diagnosis” were input with the series mean method.

|  | **Univariable** | | | | **Multivariable** | | | |
| --- | --- | --- | --- | --- | --- | --- | --- | --- |
|  | **p value** | **HR** | **95% CI** | | **p value** | **HR** | **95% CI** | |
|  |  |  | **Lower** | **Upper** |  |  | **Lower** | **Upper** |
| **Sex** |  |  |  |  |  |  |  |  |
| Female | - | - | - | - | - | - | - | - |
| Male | 0.07 | 1.103 | 0.992 | 1.227 | 0.96 | 1.003 | 0.900 | 1.117 |
| **Age** | <0.0001 | 1.037 | 1.033 | 1.041 | <0.0001 | 1.037 | 1.032 | 1.042 |
| **Comorbidities** |  |  |  |  |  |  |  |  |
| 0-1 comorbidities | - | - | - | - | - | - | - | - |
| 2+ comorbidities | <0.0001 | 1.921 | 1.729 | 2.134 | <0.0001 | 1.250 | 1.122 | 1.392 |
| **Baseline malignancy** |  |  |  |  |  |  |  |  |
| Hodgkin lymphoma | - | - | - | - | - | - | - | - |
| Chronic lymphoid leukemia | <0.0001 | 2.634 | 1.752 | 3.958 | 0.50 | 0.867 | 0.574 | 1.311 |
| Acute leukemia | <0.0001 | 2.815 | 1.883 | 4.207 | 0.08 | 1.435 | 0.959 | 2.147 |
| Non-Hodgkin lymphoma | 0.00014 | 2.157 | 1.452 | 3.206 | 0.49 | 0.870 | 0.584 | 1.296 |
| Chronic myeloid malignancies | 0.12 | 1.410 | 0.910 | 2.185 | 0.18 | 0.741 | 0.475 | 1.154 |
| Plasma cell disorders | <0.0001 | 2.502 | 1.672 | 3.742 | 0.71 | 1.081 | 0.720 | 1.622 |
| Myelodysplastic syndrome | <0.0001 | 3.163 | 2.071 | 4.832 | 0.95 | 0.985 | 0.639 | 1.517 |
| Hairy cell leukemia | 0.02 | 2.430 | 1.172 | 5.039 | 0.51 | 0.783 | 0.377 | 1.626 |
| **Malignancy status at COVID-19 diagnosis** |  |  |  |  |  |  |  |  |
| Controlled malignancy | - | - | - | - | - | - | - | - |
| Stable malignancy | <0.0001 | 1.390 | 1.184 | 1.633 | 0.23 | 1.111 | 0.937 | 1.317 |
| Active malignancy | <0.0001 | 2.731 | 2.418 | 3.085 | <0.0001 | 1.833 | 1.619 | 2.077 |
| Unknown | <0.0001 | 3.589 | 2.855 | 4.512 | <0.0001 | 2.094 | 1.660 | 2.642 |
| **Vaccine doses before COVID-19** |  |  |  |  |  |  |  |  |
| Not vaccinated | - | - | - | - | - | - | - | - |
| 1-2 doses | <0.0001 | 0.505 | 0.423 | 0.604 | 0.00088 | 0.687 | 0.551 | 0.857 |
| 3+ doses | <0.0001 | 0.286 | 0.234 | 0.351 | <0.0001 | 0.455 | 0.353 | 0.586 |
| **COVID-19 diagnosis** |  |  |  |  |  |  |  |  |
| January-June 2020 | - | - | - | - | - | - | - | - |
| July-December 2020 | <0.0001 | 0.577 | 0.506 | 0.658 | <0.0001 | 0.804 | 0.703 | 0.919 |
| January-June 2021 | <0.0001 | 0.616 | 0.515 | 0.737 | 0.014 | 0.796 | 0.661 | 0.957 |
| July-December 2021 | <0.0001 | 0.465 | 0.385 | 0.562 | 0.55 | 0.933 | 0.741 | 1.173 |
| January-June 2022 | <0.0001 | 0.321 | 0.272 | 0.379 | 0.07 | 0.827 | 0.675 | 1.015 |
| July-December 2022 | <0.0001 | 0.131 | 0.088 | 0.194 | <0.0001 | 0.424 | 0.280 | 0.642 |
| **COVID-19 symptoms at onset** |  |  |  |  |  |  |  |  |
| Screening | - | - | - | - | - | - | - | - |
| Extrapulmonary only | 0.48 | 0.927 | 0.750 | 1.145 | 0.13 | 0.847 | 0.684 | 1.048 |
| Extrapulmonary + pulmonary | <0.0001 | 2.122 | 1.787 | 2.520 | 0.10 | 1.163 | 0.973 | 1.390 |
| Pulmonary only | <0.0001 | 2.149 | 1.822 | 2.536 | 0.0026 | 1.300 | 1.096 | 1.542 |
| **Stay during COVID-19 episode** |  |  |  |  |  |  |  |  |
| Home | - | - | - | - | - | - | - | - |
| Hospital. no ICU | <0.0001 | 22.385 | 15.359 | 32.625 | <0.0001 | 12.821 | 8.761 | 18.764 |
| Hospital. ICU | <0.0001 | 57.199 | 39.137 | 83.596 | <0.0001 | 33.820 | 23.008 | 49.714 |

COVID-19, coronavirus disease 2019; HR, hazard ratio; ICU, intensive care unit

**Supplementary table 8.** Factors associated with mortality in EPICOVIDEHA 2020 – 2022 patients. Sensitivy analysis with missing values in “Days from COVID-19 diagnosis” were input with the linear interpolation method.

|  | **Univariable** | | | | **Multivariable** | | | |
| --- | --- | --- | --- | --- | --- | --- | --- | --- |
|  | **p value** | **HR** | **95% CI** | | **p value** | **HR** | **95% CI** | |
|  |  |  | **Lower** | **Upper** |  |  | **Lower** | **Upper** |
| **Sex** |  |  |  |  |  |  |  |  |
| Female | - | - | - | - | - | - | - | - |
| Male | 0.08 | 1.102 | 0.990 | 1.225 | 0.97 | 1.002 | 0.900 | 1.116 |
| **Age** | <0.0001 | 1.037 | 1.033 | 1.041 | <0.0001 | 1.037 | 1.032 | 1.042 |
| **Comorbidities** |  |  |  |  |  |  |  |  |
| 0-1 comorbidities | - | - | - | - | - | - | - | - |
| 2+ comorbidities | <0.0001 | 1.919 | 1.728 | 2.132 | <0.0001 | 1.247 | 1.119 | 1.389 |
| **Baseline malignancy** |  |  |  |  |  |  |  |  |
| Hodgkin lymphoma | - | - | - | - | - | - | - | - |
| Chronic lymphoid leukemia | <0.0001 | 2.636 | 1.754 | 3.962 | 0.51 | 0.871 | 0.576 | 1.316 |
| Acute leukemia | <0.0001 | 2.816 | 1.884 | 4.209 | 0.08 | 1.433 | 0.957 | 2.144 |
| Non-Hodgkin lymphoma | 0.00015 | 2.155 | 1.450 | 3.203 | 0.49 | 0.869 | 0.583 | 1.295 |
| Chronic myeloid malignancies | 0.12 | 1.413 | 0.912 | 2.190 | 0.19 | 0.744 | 0.477 | 1.159 |
| Plasma cell disorders | <0.0001 | 2.502 | 1.673 | 3.744 | 0.71 | 1.081 | 0.720 | 1.621 |
| Myelodysplastic syndrome | <0.0001 | 3.164 | 2.071 | 4.833 | 0.95 | 0.985 | 0.640 | 1.517 |
| Hairy cell leukemia | 0.017 | 2.424 | 1.169 | 5.026 | 0.51 | 0.782 | 0.376 | 1.624 |
| **Malignancy status at COVID-19 diagnosis** |  |  |  |  |  |  |  |  |
| Controlled malignancy | - | - | - | - | - | - | - | - |
| Stable malignancy | <0.0001 | 1.392 | 1.185 | 1.635 | 0.22 | 1.113 | 0.939 | 1.319 |
| Active malignancy | <0.0001 | 2.731 | 2.418 | 3.085 | <0.0001 | 1.833 | 1.618 | 2.076 |
| Unknown | <0.0001 | 3.610 | 2.872 | 4.539 | <0.0001 | 2.106 | 1.670 | 2.657 |
| **Vaccine doses before COVID-19** |  |  |  |  |  |  |  |  |
| Not vaccinated | - | - | - | - | - | - | - | - |
| 1-2 doses | <0.0001 | 0.504 | 0.422 | 0.602 | <0.0001 | 0.684 | 0.549 | 0.854 |
| 3+ doses | <0.0001 | 0.286 | 0.233 | 0.350 | 0.00076 | 0.453 | 0.351 | 0.584 |
| **COVID-19 diagnosis** |  |  |  |  |  |  |  |  |
| January-June 2020 | - | - | - | - | - | - | - | - |
| July-December 2020 | <0.0001 | 0.577 | 0.506 | 0.658 | 0.0013 | 0.803 | 0.702 | 0.918 |
| January-June 2021 | <0.0001 | 0.617 | 0.516 | 0.738 | 0.016 | 0.796 | 0.661 | 0.958 |
| July-December 2021 | <0.0001 | 0.467 | 0.387 | 0.565 | 0.57 | 0.935 | 0.743 | 1.177 |
| January-June 2022 | <0.0001 | 0.322 | 0.272 | 0.380 | 0.07 | 0.830 | 0.677 | 1.018 |
| July-December 2022 | <0.0001 | 0.132 | 0.089 | 0.195 | <0.0001 | 0.426 | 0.281 | 0.645 |
| **COVID-19 symptoms at onset** |  |  |  |  |  |  |  |  |
| Screening | - | - | - | - | - | - | - | - |
| Extrapulmonary only | 0.48 | 0.927 | 0.751 | 1.146 | 0.13 | 0.847 | 0.685 | 1.049 |
| Extrapulmonary + pulmonary | <0.0001 | 2.126 | 1.791 | 2.525 | 0.09 | 1.166 | 0.975 | 1.393 |
| Pulmonary only | <0.0001 | 2.149 | 1.821 | 2.536 | 0.0026 | 1.300 | 1.096 | 1.541 |
| **Stay during COVID-19 episode** |  |  |  |  |  |  |  |  |
| Home | - | - | - | - | - | - | - | - |
| Hospital. no ICU | <0.0001 | 22.375 | 15.352 | 32.611 | <0.0001 | 12.795 | 8.742 | 18.725 |
| Hospital. ICU | <0.0001 | 57.124 | 39.086 | 83.486 | <0.0001 | 33.758 | 22.965 | 49.622 |

COVID-19, coronavirus disease 2019; HR, hazard ratio; ICU, intensive care unit

**Supplementary table 9.** Factors associated with mortality in EPICOVIDEHA 2020 – 2022 patients. Sensitivy analysis with missing values in “Days from COVID-19 diagnosis” were input with the linear trend at point method.

|  | **Univariable** | | | | **Multivariable** | | | |
| --- | --- | --- | --- | --- | --- | --- | --- | --- |
|  | **p value** | **HR** | **95% CI** | | **p value** | **HR** | **95% CI** | |
|  |  |  | **Lower** | **Upper** |  |  | **Lower** | **Upper** |
| **Sex** |  |  |  |  |  |  |  |  |
| Female | - | - | - | - | - | - | - | - |
| Male | 0.07 | 1.104 | 0.992 | 1.228 | 0.956 | 1.003 | 0.901 | 1.117 |
| **Age** | <0.0001 | 1.037 | 1.033 | 1.041 | <0.0001 | 1.037 | 1.032 | 1.042 |
| **Comorbidities** |  |  |  |  |  |  |  |  |
| 0-1 comorbidities | - | - | - | - | - | - | - | - |
| 2+ comorbidities | <0.0001 | 1.922 | 1.730 | 2.135 | <0.0001 | 1.251 | 1.122 | 1.393 |
| **Baseline malignancy** |  |  |  |  |  |  |  |  |
| Hodgkin lymphoma | - | - | - | - | - | - | - | - |
| Chronic lymphoid leukemia | <0.0001 | 2.633 | 1.752 | 3.957 | 0.50 | 0.866 | 0.573 | 1.309 |
| Acute leukemia | <0.0001 | 2.814 | 1.883 | 4.206 | 0.08 | 1.435 | 0.959 | 2.148 |
| Non-Hodgkin lymphoma | 0.00014 | 2.158 | 1.452 | 3.207 | 0.50 | 0.870 | 0.584 | 1.297 |
| Chronic myeloid malignancies | 0.13 | 1.409 | 0.909 | 2.183 | 0.18 | 0.740 | 0.475 | 1.152 |
| Plasma cell disorders | <0.0001 | 2.501 | 1.672 | 3.742 | 0.71 | 1.081 | 0.720 | 1.622 |
| Myelodysplastic syndrome | <0.0001 | 3.163 | 2.071 | 4.832 | 0.95 | 0.985 | 0.639 | 1.517 |
| Hairy cell leukemia | 0.017 | 2.432 | 1.173 | 5.043 | 0.51 | 0.783 | 0.377 | 1.627 |
| **Malignancy status at COVID-19 diagnosis** |  |  |  |  |  |  |  |  |
| Controlled malignancy | - | - | - | - | - | - | - | - |
| Stable malignancy | <0.0001 | 1.390 | 1.183 | 1.632 | 0.23 | 1.110 | 0.937 | 1.316 |
| Active malignancy | <0.0001 | 2.731 | 2.417 | 3.084 | <0.0001 | 1.834 | 1.619 | 2.077 |
| Unknown | <0.0001 | 3.582 | 2.849 | 4.503 | <0.0001 | 2.090 | 1.657 | 2.636 |
| **Vaccine doses before COVID-19** |  |  |  |  |  |  |  |  |
| Not vaccinated | - | - | - | - | - | - | - | - |
| 1-2 doses | <0.0001 | 0.506 | 0.424 | 0.604 | 0.00092 | 0.688 | 0.552 | 0.859 |
| 3+ doses | <0.0001 | 0.286 | 0.234 | 0.351 | <0.0001 | 0.455 | 0.353 | 0.587 |
| **COVID-19 diagnosis** |  |  |  |  |  |  |  |  |
| January-June 2020 | - | - | - | - | - | - | - | - |
| July-December 2020 | <0.0001 | 0.577 | 0.506 | 0.658 | 0.00014 | 0.804 | 0.704 | 0.920 |
| January-June 2021 | <0.0001 | 0.616 | 0.515 | 0.737 | 0.015 | 0.795 | 0.661 | 0.957 |
| July-December 2021 | <0.0001 | 0.464 | 0.384 | 0.561 | 0.55 | 0.932 | 0.740 | 1.172 |
| January-June 2022 | <0.0001 | 0.321 | 0.272 | 0.379 | 0.07 | 0.826 | 0.674 | 1.013 |
| July-December 2022 | <0.0001 | 0.131 | 0.088 | 0.194 | <0.0001 | 0.423 | 0.280 | 0.641 |
| **COVID-19 symptoms at onset** |  |  |  |  |  |  |  |  |
| Screening | - | - | - | - | - | - | - | - |
| Extrapulmonary only | 0.48 | 0.927 | 0.750 | 1.145 | 0.13 | 0.847 | 0.684 | 1.048 |
| Extrapulmonary + pulmonary | <0.0001 | 2.121 | 1.786 | 2.518 | 0.10 | 1.162 | 0.972 | 1.389 |
| Pulmonary only | <0.0001 | 2.149 | 1.821 | 2.536 | 0.0026 | 1.300 | 1.096 | 1.542 |
| **Stay during COVID-19 episode** |  |  |  |  |  |  |  |  |
| Home | - | - | - | - | - | - | - | - |
| Hospital. no ICU | <0.0001 | 22.387 | 15.360 | 32.628 | <0.0001 | 12.830 | 8.767 | 18.777 |
| Hospital. ICU | <0.0001 | 57.218 | 39.150 | 83.624 | <0.0001 | 33.838 | 23.020 | 49.740 |

COVID-19, coronavirus disease 2019; HR, hazard ratio; ICU, intensive care unit

**SUPPLEMENTARY FIGURES**

**Supplementary figure 1.** EPICOVIDEHA 2020 – 2022 patients: geographical distribution

**
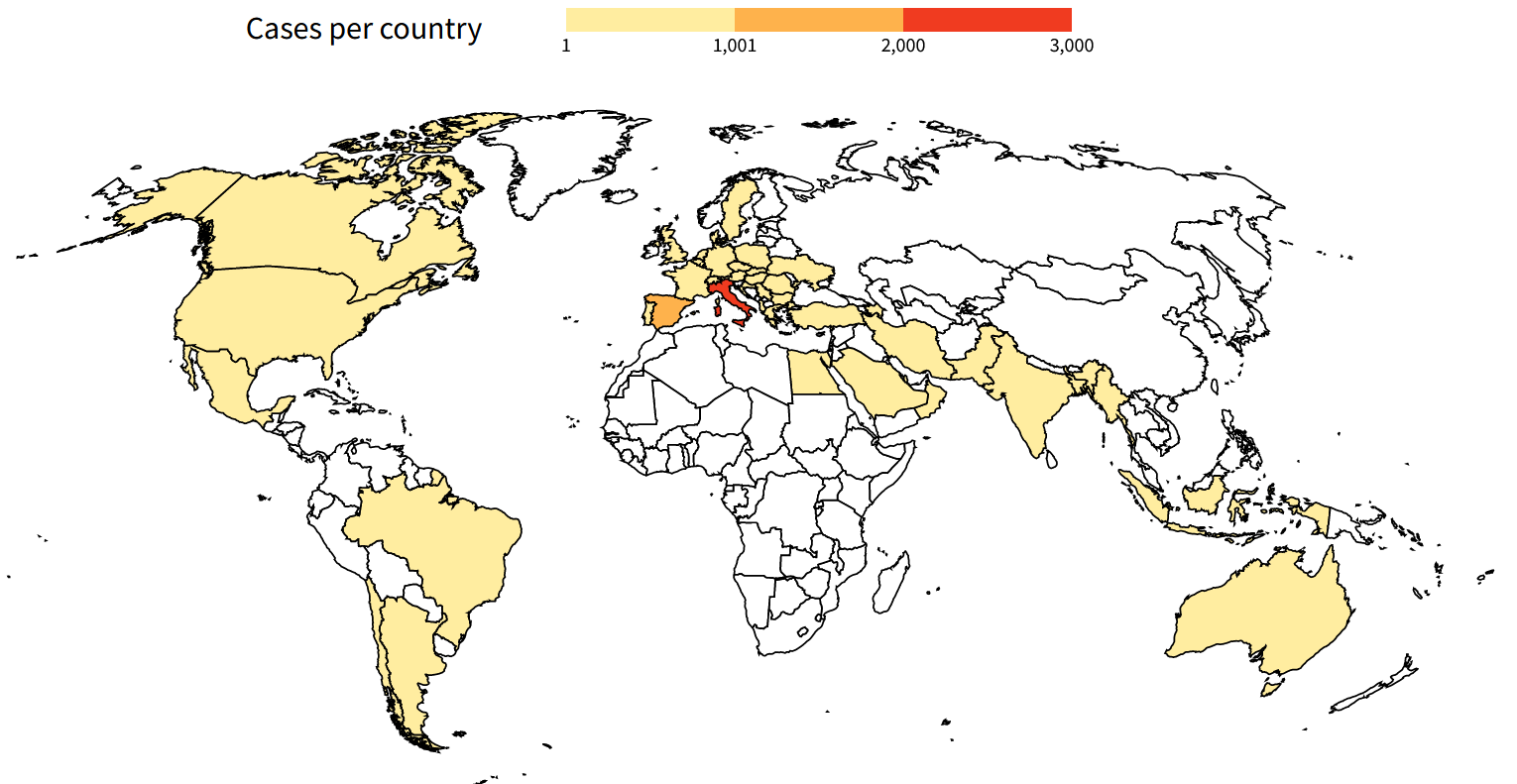
**

Participants were being treated in centers from Italy (n=2294), Spain (n=1511), Czech Republic (n=581), France (n=437), Netherlands (n=356), Serbia (n=347), Germany (n=319), Denmark (n=263), Belgium (n=261), Croatia (n=260), Portugal (n=251), Turkey (n=249), Hungary (n=202), United Kingdom (n=159), Egypt (n=154), Brazil (n=134), Pakistan (n=131), Austria (n=118), Sweden (n=109), Greece (n=89), Argentina (n=74), United States (n=70), North Macedonia (n=65), Poland (n=61), Qatar (n=52), Switzerland (n=47), Saudi Arabia (n=40), Oman (n=24), Bulgaria (n=20), Indonesia (n=17), Chile (n=15), Slovakia (n=12), Azerbaijan and Romania (n=7, each), Iran and Myanmar (n=6, each), Albania (n=5), Hong Kong S.A.R. (n=4), Australia, Canada, and Mexico (n=2, each), Bangladesh, India, Singapore, and Ukraine (n=1, each).

**Supplementary figure 2.** EPICOVIDEHA 2020 – 2022 patients: prevalence per underlying comorbidities, baseline malignancy, and last baseline malignancy treatment

**S2A) 2020 – 2022 prevalence per underlying comorbidities**

**
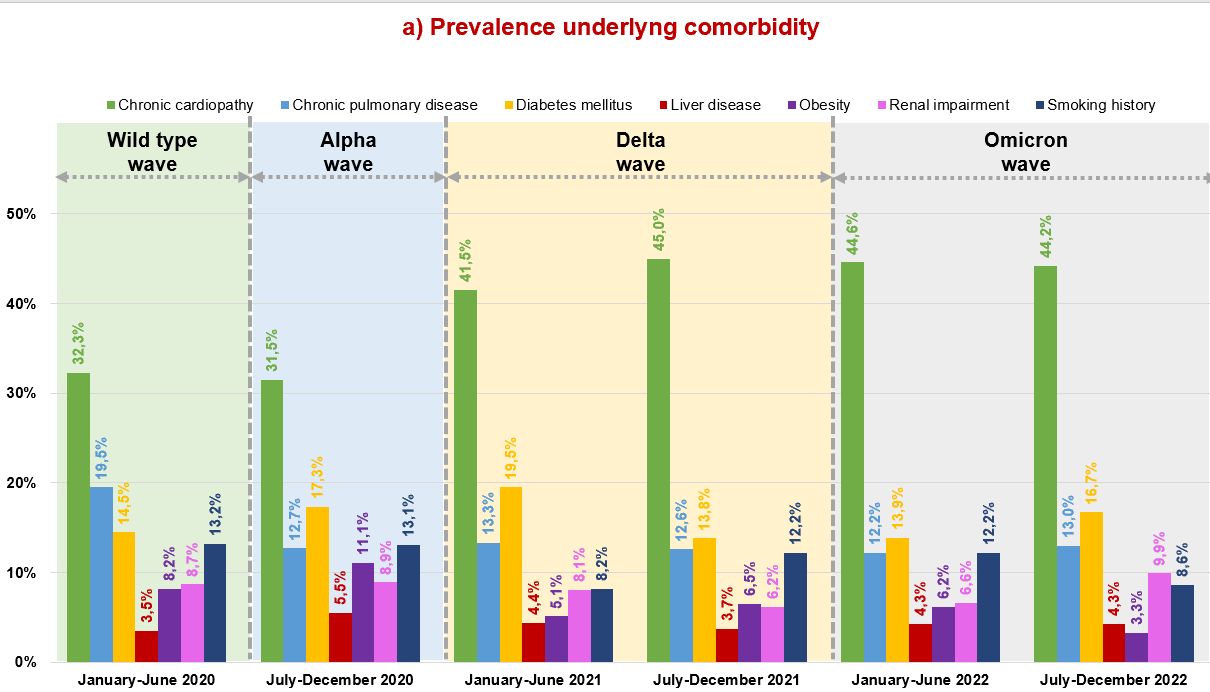
**

**S2B) 2020 – 2022 prevalence per baseline malignancy**


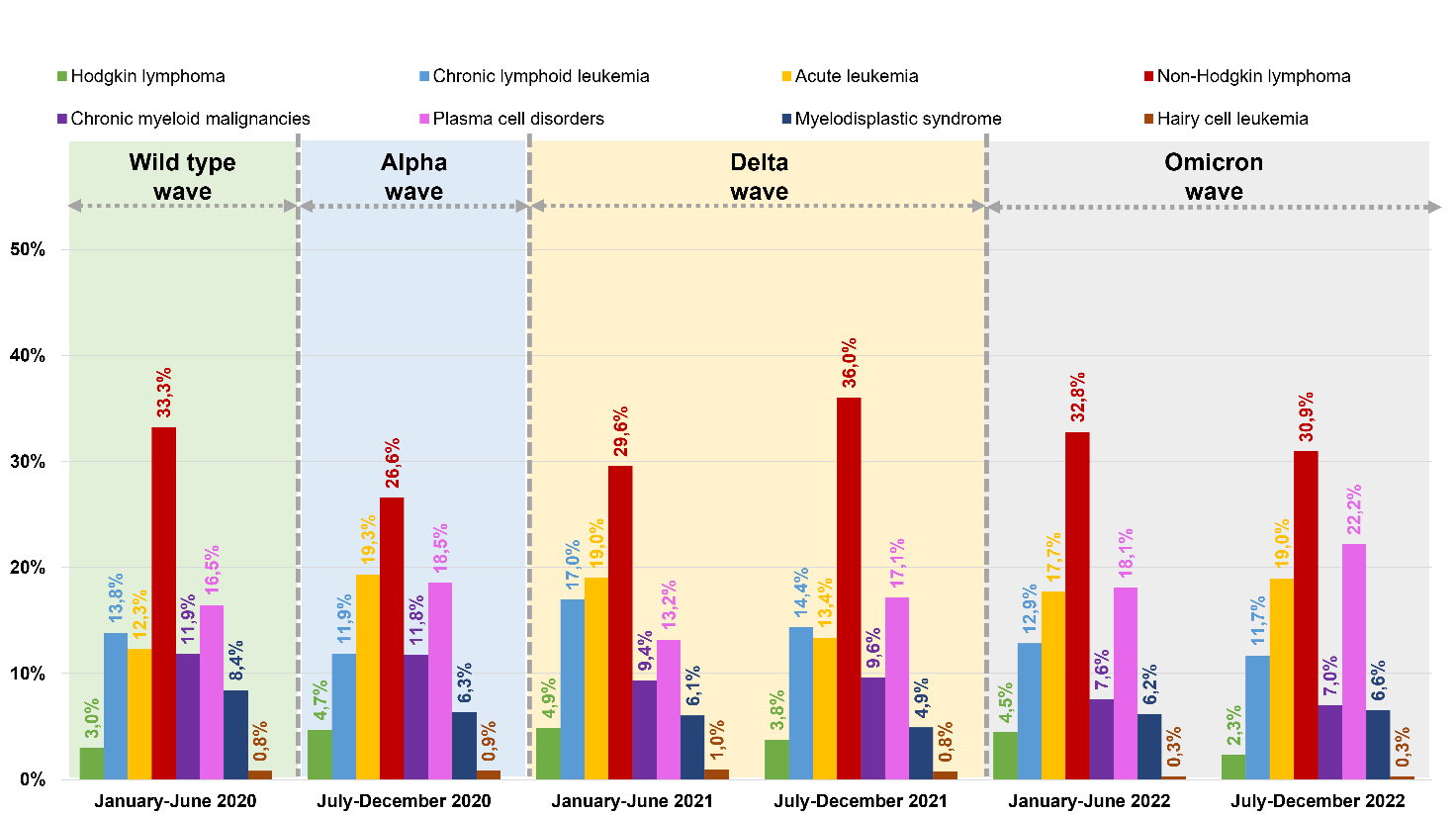


**S2C) 2020 – 2022 prevalence per last baseline malignancy treatment**

**
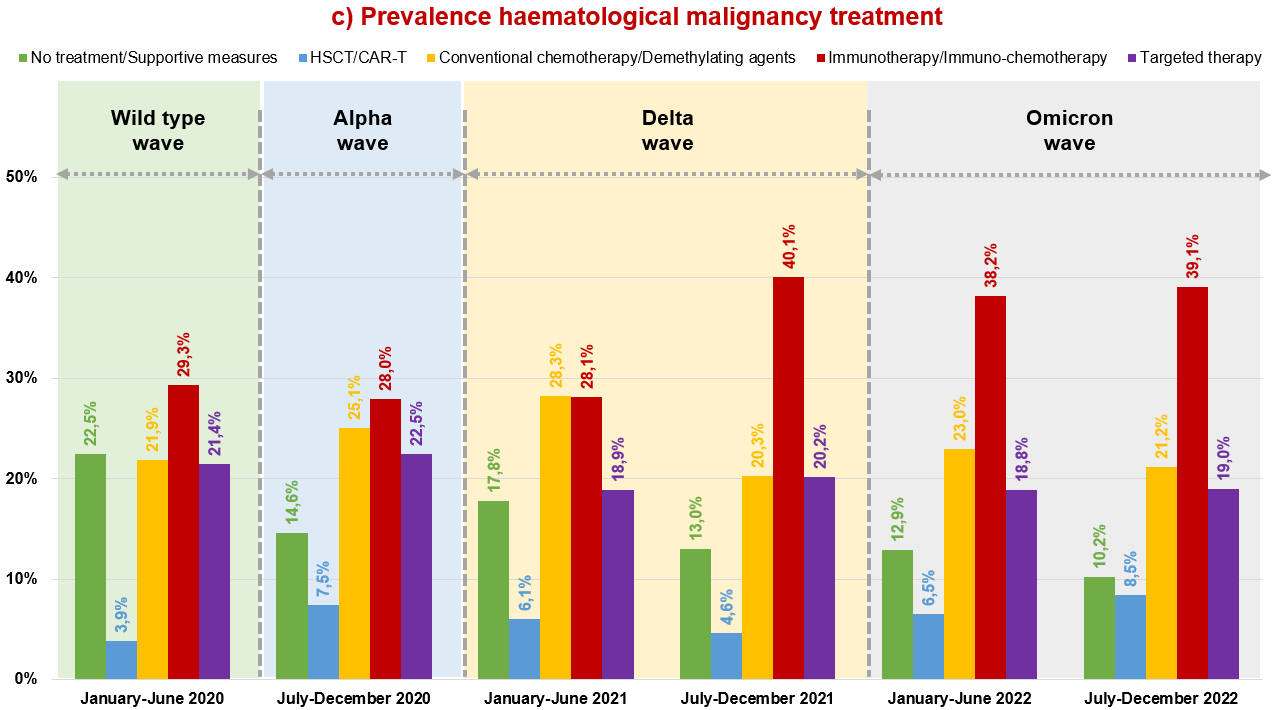
**

CAR-T, chimeric antigen T cell receptors; HSCT, hematopoietic stem cell transplantation

**Supplementary figure 3.** EPICOVIDEHA 2020 – 2022 patients: survival probability per underlying malignancy and place of COVID-19 episode stay

**S3A) 2020 – 2022 survival probability per baseline malignancy and year**


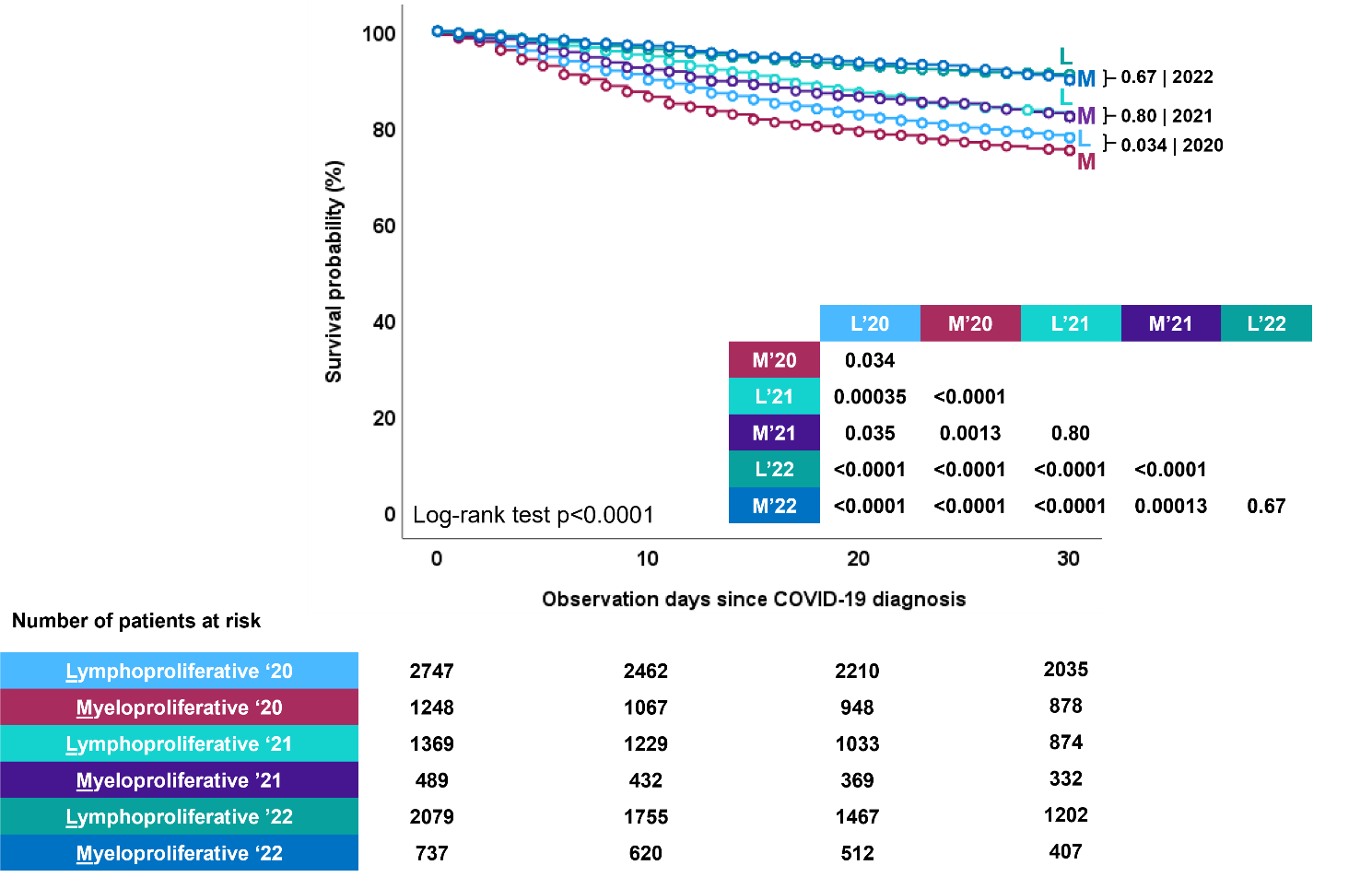


**S3B) 2020 – 2022 survival probability per stay during COVID-19 episode and year**


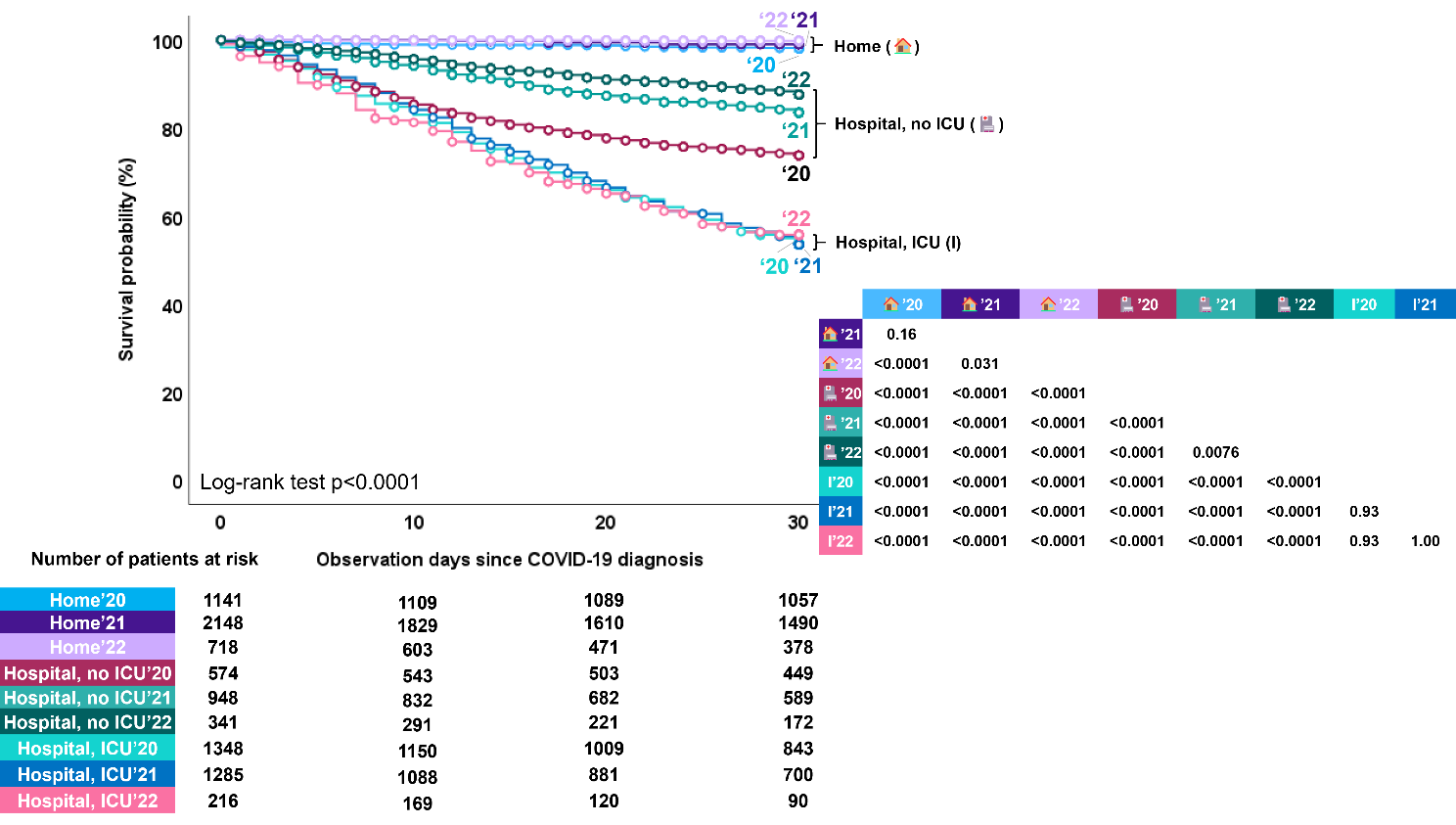

Supplement: Supplementary Figs. S1–S3 and Tables S1–S9 [file mmc1.docx]
