# Supplementary material for: Decoding the historical tale: COVID-19 impact on haematological malignancy patients—EPICOVIDEHA insights from 2020 to 2022
Source: eClinicalMedicine. 2024 Mar 18;71:102553. doi: 10.1016/j.eclinm.2024.102553 (PMC10963230; doi:10.1016/j.eclinm.2024.102553)

Supplementary figure 5. Martingale residuals plot of the variables presented in the Cox regression analysis provided in Table 2.

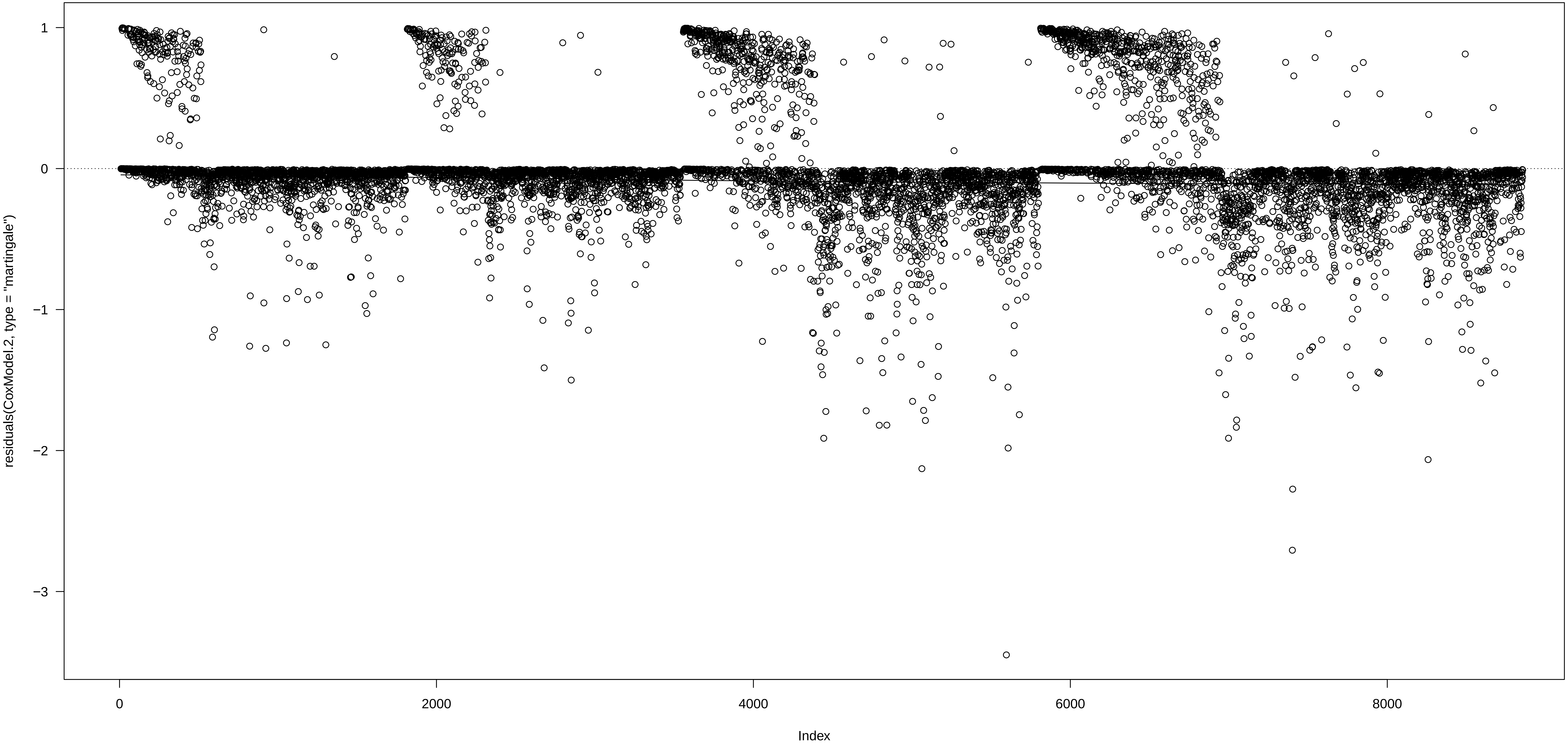

Supplement: Supplementary Fig. S5 [file mmc3.pdf]
