# Supplementary material for: Decoding the historical tale: COVID-19 impact on haematological malignancy patients—EPICOVIDEHA insights from 2020 to 2022
Source: eClinicalMedicine. 2024 Mar 18;71:102553. doi: 10.1016/j.eclinm.2024.102553 (PMC10963230; doi:10.1016/j.eclinm.2024.102553)
Supplement: Supplementary Fig. S6 [file mmc4.pdf]

Supplementary figure 6. Proportional hazard evaluation of the variables presented in the Cox regression analysis provided in Table 2.

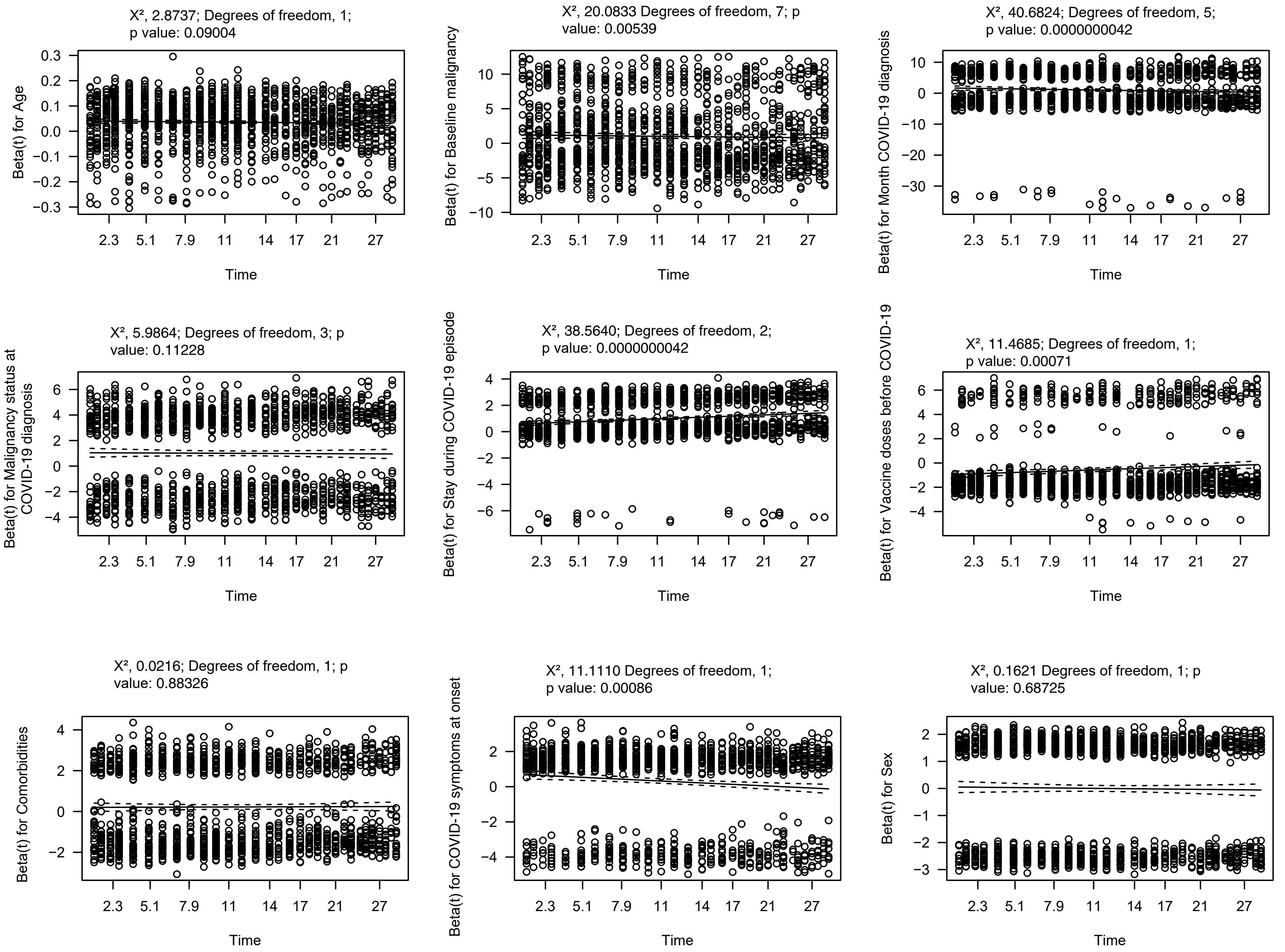

In this sensitivity analysis answer options have been grouped as follows : Vaccination doses before COVID-19 (No vaccination and At least 1 dose);and COVID-19 symptoms at onset (No pulmonary involvement and Pulmonary involvement)
