# Supplementary material for: Decoding the historical tale: COVID-19 impact on haematological malignancy patients—EPICOVIDEHA insights from 2020 to 2022
Source: eClinicalMedicine. 2024 Mar 18;71:102553. doi: 10.1016/j.eclinm.2024.102553 (PMC10963230; doi:10.1016/j.eclinm.2024.102553)

Supplementary figure 7. Martingale residuals plot of the variables presented in the Cox regression analysis provided in Table 2.

In this sensitivity analysis answer options have been grouped as follows : Vaccination doses before COVID-19 (No vaccination and At least 1 dose); Month COVID-19 diagnosis (January-June 2020, July-Dcember 2020, January-December 2021, and January-December 2022); and COVID-19 symptoms at onset (No pulmonary involvement and Pulmonary involvement)

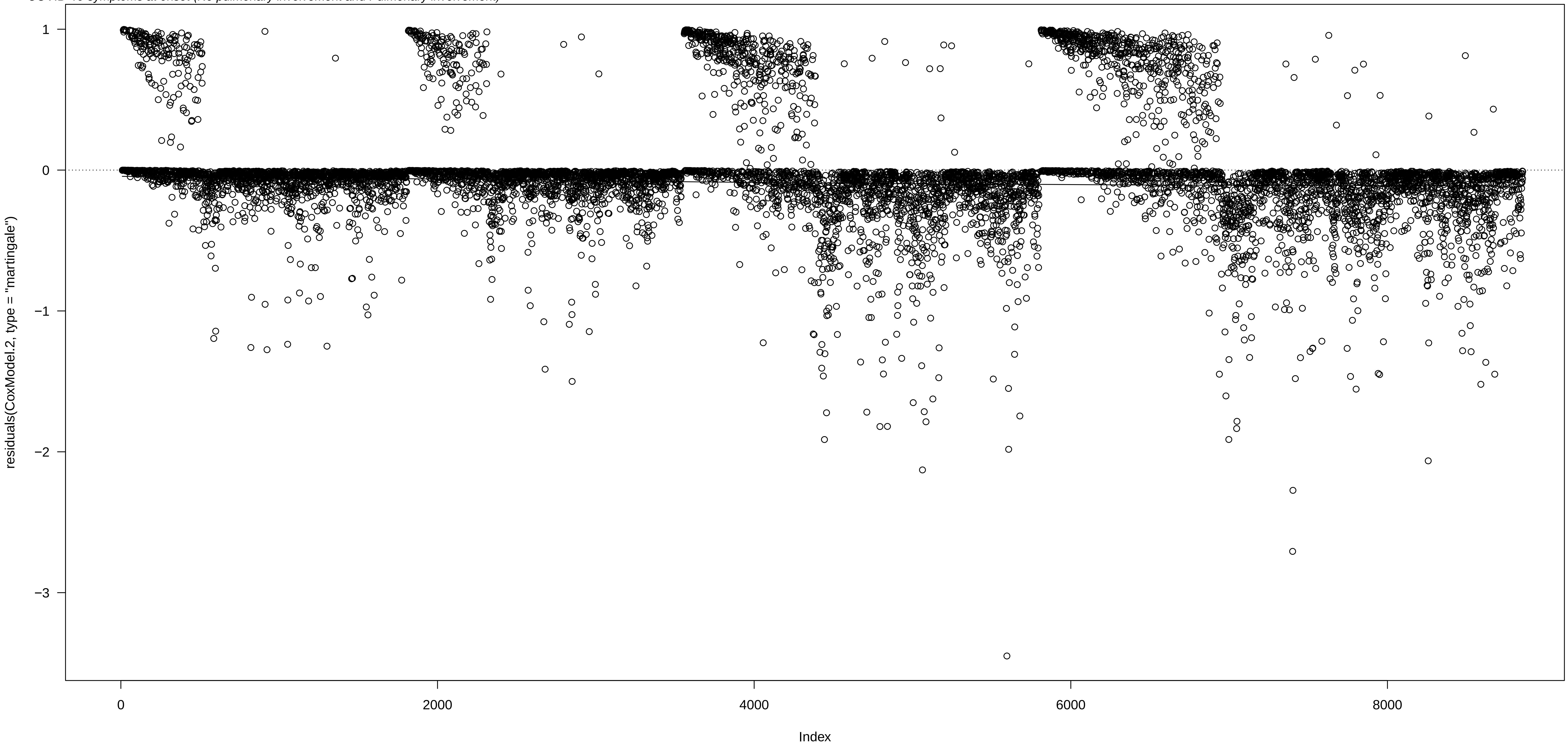

Supplement: Supplementary Fig. S7 [file mmc5.pdf]
