# Supplementary figures and images for: Decoding the historical tale: COVID-19 impact on haematological malignancy patients—EPICOVIDEHA insights from 2020 to 2022
Source: eClinicalMedicine. 2024 Mar 18;71:102553. doi: 10.1016/j.eclinm.2024.102553 (PMC10963230; doi:10.1016/j.eclinm.2024.102553)

**Supplementary figure 8.** 2020 – 2022 survival probability per semester (Y axis 70% to 100%)

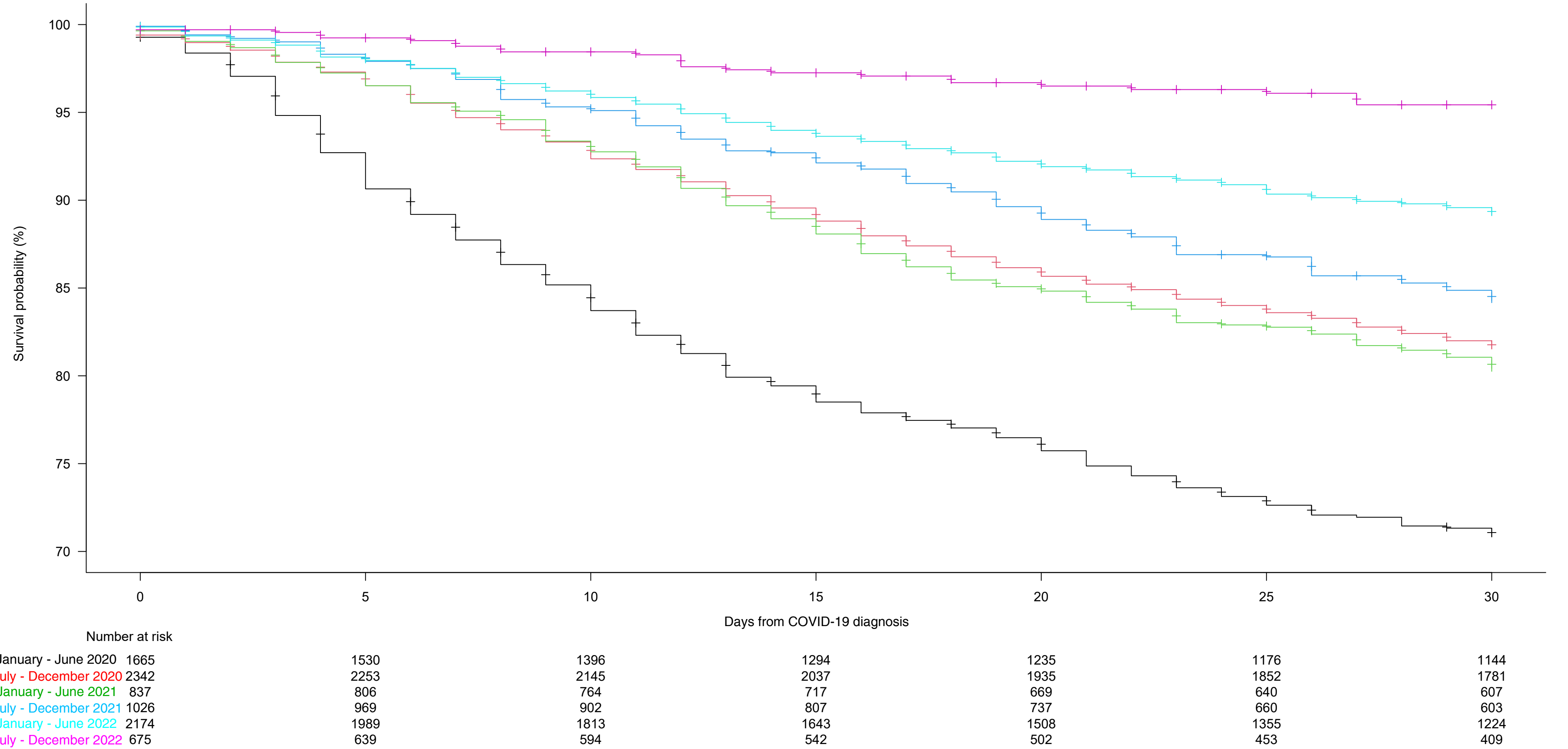

Supplement: Supplementary Fig. S8 [file mmc6.pdf]

**Supplementary figure 9.** 2020 – 2022 survival probability per vaccine doses before COVID-19 (Y axis 70% to 100%)

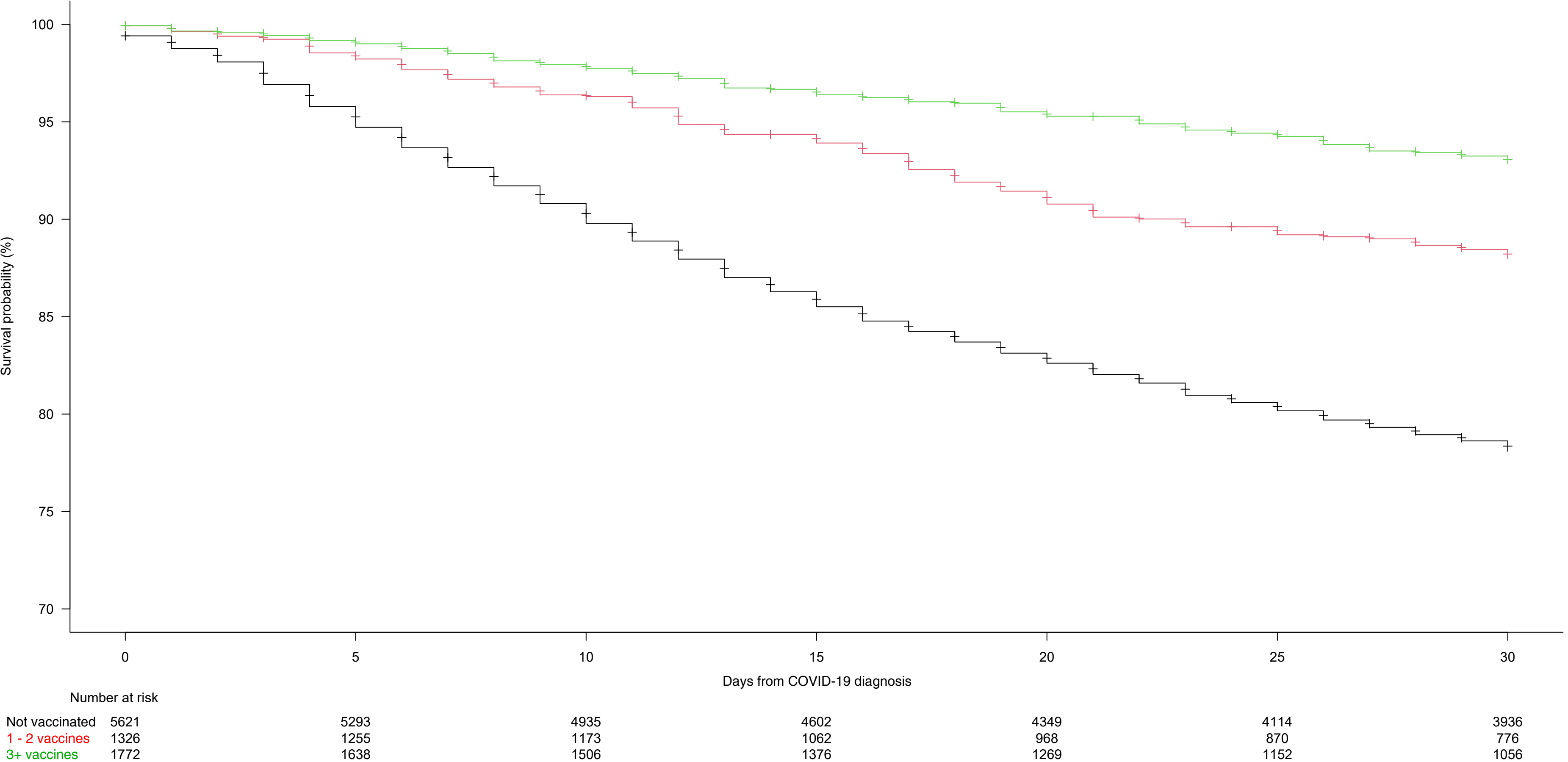

Supplement: Supplementary Fig. S9 [file mmc7.pdf]
