# Supplementary material for: Decoding the historical tale: COVID-19 impact on haematological malignancy patients—EPICOVIDEHA insights from 2020 to 2022
Source: eClinicalMedicine. 2024 Mar 18;71:102553. doi: 10.1016/j.eclinm.2024.102553 (PMC10963230; doi:10.1016/j.eclinm.2024.102553)
Supplement: Supplementary Fig. S10 [file mmc8.pdf]

Supplementary figure 10. 2020 – 2022 survival probability per vaccine doses before COVID-19 and COVID-19 treatment, 0 vaccines *(Y axis 70% to 100%)*

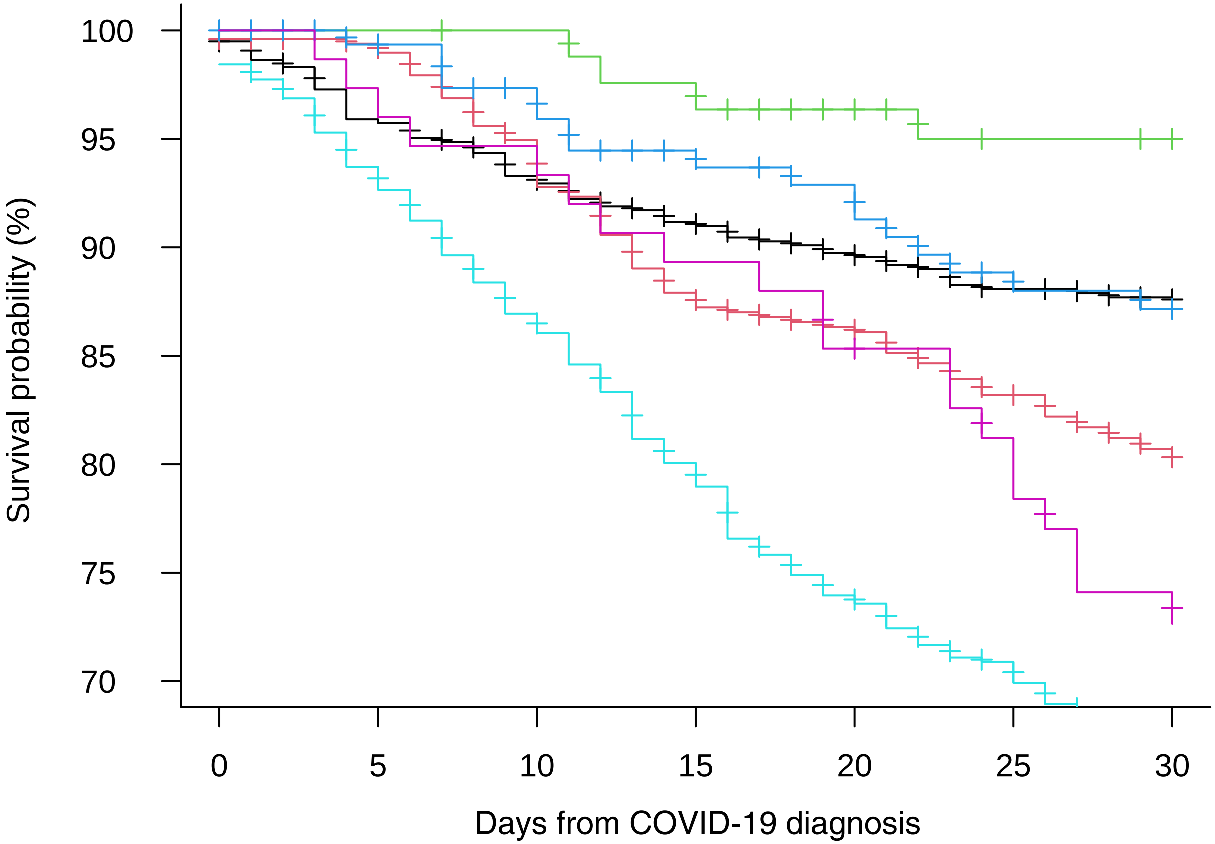

Number at risk

	0	5	10	15	20	25	30
No treatment	594	557	532	508	494	472	456
AVs ± plasma ± corticosteroids	495	479	438	389	371	338	320
AVs ± MoABs ± plasma ± corticosteroids	84	84	83	80	74	67	66
MoABs ± plasma ± corticosteroids	164	152	137	121	116	106	101
Corticosteroids	575	531	483	438	391	365	336
Plasma	75	73	71	67	63	58	51
