# Supplementary material for: Decoding the historical tale: COVID-19 impact on haematological malignancy patients—EPICOVIDEHA insights from 2020 to 2022
Source: eClinicalMedicine. 2024 Mar 18;71:102553. doi: 10.1016/j.eclinm.2024.102553 (PMC10963230; doi:10.1016/j.eclinm.2024.102553)
Supplement: Supplementary Fig. S11 [file mmc9.pdf]

Supplementary figure 11. 2020 – 2022 survival probability per vaccine doses before COVID-19 and COVID-19 treatment, 1-2 vaccines *(Y axis 70% to 100%)*

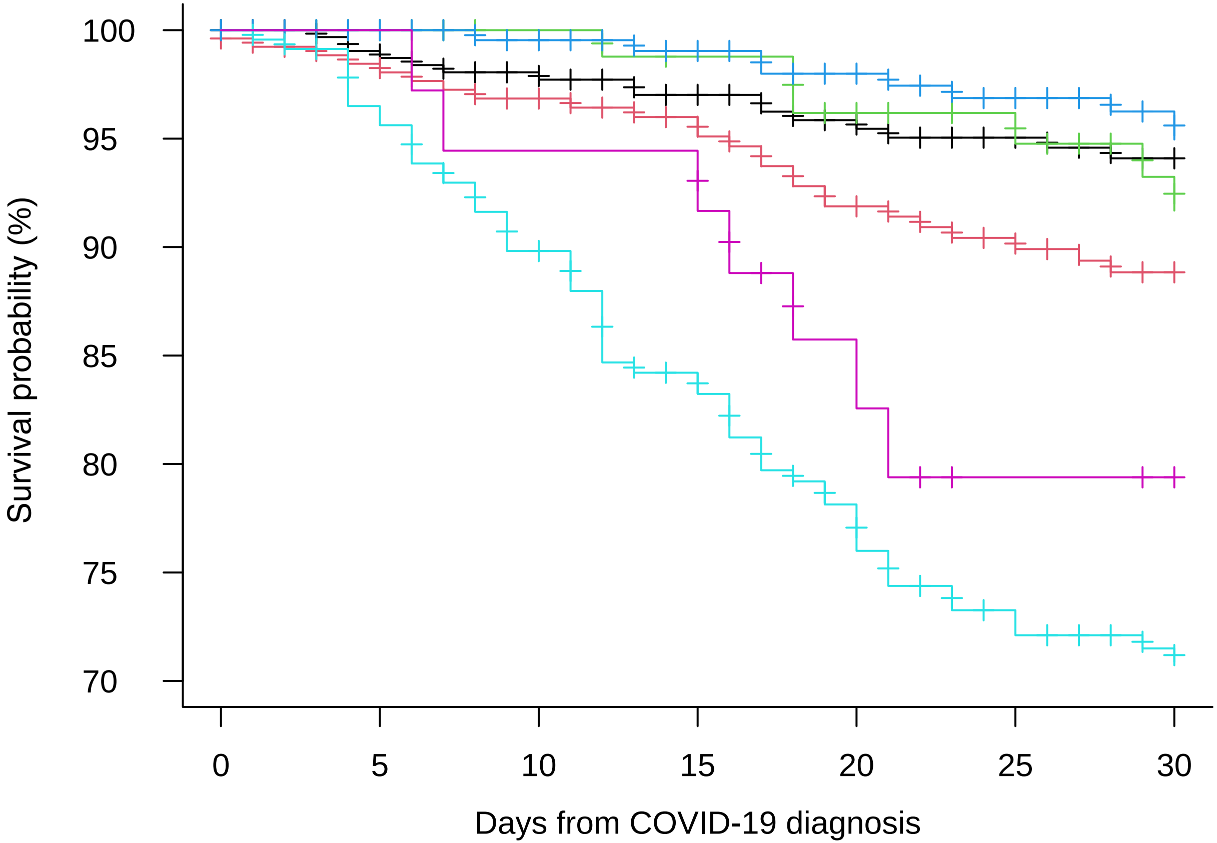

Number at risk

	0	5	10	15	20	25	30
No treatment	327	308	289	259	240	214	176
AVs ± plasma ± corticosteroids	262	249	236	215	195	175	161
AVs ± MoABs ± plasma ± corticosteroids	88	86	82	76	71	68	60
MoABs ± plasma ± corticosteroids	238	222	208	192	181	162	149
Corticosteroids	231	219	196	172	146	127	115
Plasma	36	36	34	34	27	23	22
