# Supplementary material for: Decoding the historical tale: COVID-19 impact on haematological malignancy patients—EPICOVIDEHA insights from 2020 to 2022
Source: eClinicalMedicine. 2024 Mar 18;71:102553. doi: 10.1016/j.eclinm.2024.102553 (PMC10963230; doi:10.1016/j.eclinm.2024.102553)
Supplement: Supplementary Fig. S12 [file mmc10.pdf]

Supplementary figure 12. 2020 – 2022 survival probability per vaccine doses before COVID-19 and COVID-19 treatment, 3+ vaccines *(Y axis 70% to 100%)*

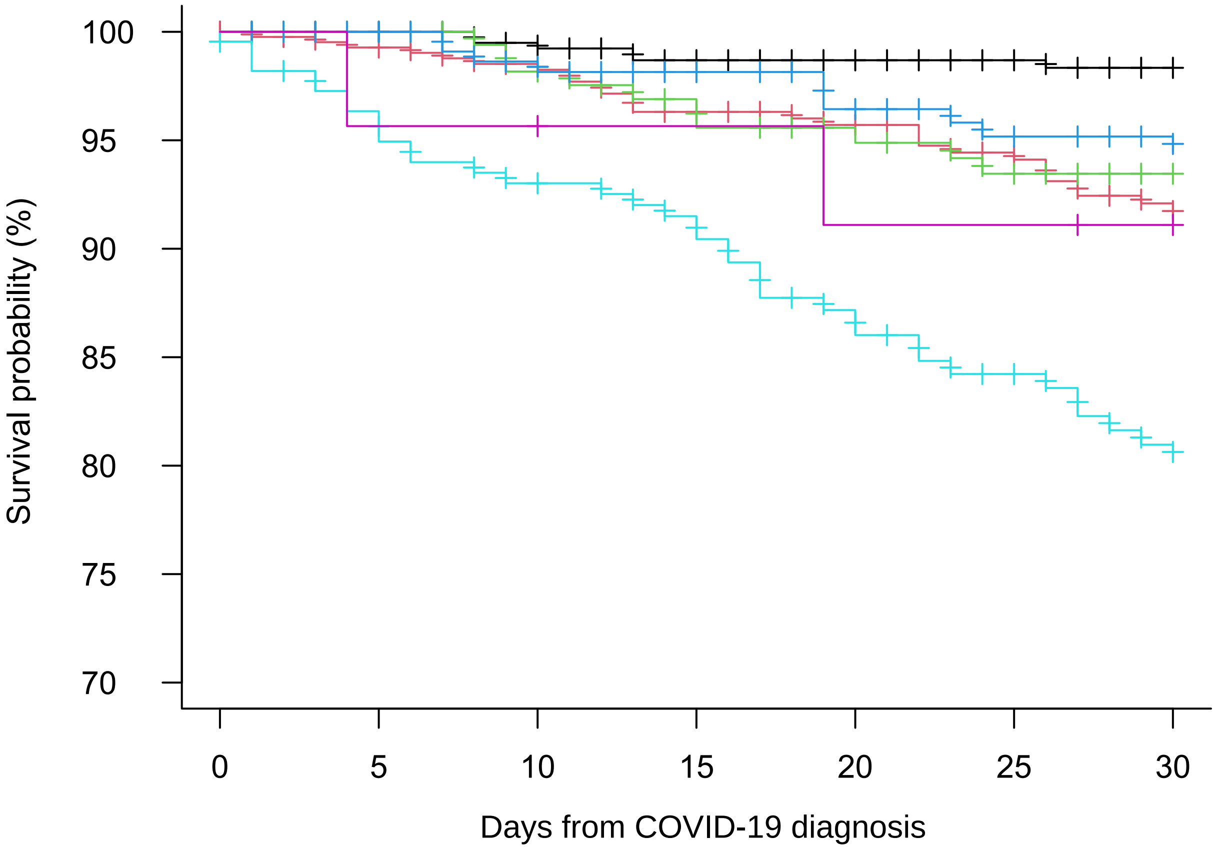

Number at risk

	0	5	10	15	20	25	30
No treatment	466	418	383	349	320	287	262
AVs ± plasma ± corticosteroids	428	403	368	331	312	290	260
AVs ± MoABs ± plasma ± corticosteroids	176	171	158	147	138	127	118
MoABs ± plasma ± corticosteroids	255	227	203	184	167	145	140
Corticosteroids	222	207	190	173	151	133	120
Plasma	23	22	22	21	20	20	19
