# Supplementary material for: Decoding the historical tale: COVID-19 impact on haematological malignancy patients—EPICOVIDEHA insights from 2020 to 2022
Source: eClinicalMedicine. 2024 Mar 18;71:102553. doi: 10.1016/j.eclinm.2024.102553 (PMC10963230; doi:10.1016/j.eclinm.2024.102553)
Supplement: R3. Collaborators [file mmc11.docx]

**Collaborators (from** on behalf of the EPICOVIDEHA registry**, to be mentioned in PubMed)**

Francisco Javier **MARTÍN-VALLEJO,** Przemyslaw **ZDZIARSKI**, Hossein **ZARRINFER**, Jana **WITTIG**, Sein **WIN**, Vivien **WAI-MAN**, Benjamín **VÍŠEK**, Donald C. **VINH**, Maria **VEHRESCHILD**, Gina **VARRICCHIO**, Panagiotis **TSIRIGOTIS**, Ana **TORRES-TIENZA**, Alina Daniela **TANASE**, Agostino **TAFURI**, Maria **STAMOULI**, Jiří **SRAMEK**, Carole **SOUSSAIN**, Ayten **SHIRINOVA**, Jörg **SCHUBERT**, Enrico **SCHALK**, Mohammad Reza **SALEHI**, Modar **SALEH**, Giorgio **ROSATI**, Elisa **ROLDÁN**, Florian **REIZINE**, Mayara **RÊGO**, Isabel **REGALADO-ARTAMENDI**, Marina **POPOVA**, Fernando **PINTO**, Laure **PHILIPPE**, Hans Martin **ORTH**, Hans-Beier **OMMEN**, Aleš **OBR**, Lucía **NÚÑEZ-MARTÍN-BUITRAGO**, Nicolas **NOËL**, Julia **NEUHANN**, Gianpaolo **NADALI**, Julia A. **NACOV**, Ana M. **MUNHOZ ALBURQUERQUE**, Maria Enza **MITRA**, Malgorzata **MIKULSKA**, Sibylle **MELLINGHOFF**, Ben **MECHTEL**, Juan-Alberto **MARTÍN-GONZÁLEZ**, Sandra **MALAK**, Jorge **LOUREIRO-AMIGO**, Lisset **LORENZO DE LA PEÑA**, Giulia **LIBERTI**, Marianne **LANDAU**, Ira **LACEJ**, Martin **KOLDITZ**, Chi Shan **KHO**, Reham Abdelaziz **KHEDR**, Meinolf **KARTHAUS**, Linda Katharina **KARLSSON**, María-Josefa **JIMÉNEZ-LORENZO**, Macarena **IZUZQUIZA**, Baerbel **HOELL-NEUGEBAUER**, Raoul **HERBRECHT**, Christopher H. **HEATH**, Fabio **GUOLO**, Jan **GROTHE**, Antonio **GIORDANO**, Sergey **GERASYMCHUK**, Ramón **GARCÍA-SANZ**, Nicole **GARCÍA-POUTÓN**, Vaneuza Araújo Moreira **FUNKE**, Monica **FUNG**, Charlotte **FLASSHOVE**, Luana **FIANCHI**, Jenna **ESSAME**, Matthias **EGGER**, Bernard **DRENOU**, Giulia **DRAGONETTI**, Maximilian **DESOLE**, Roberta **DELLA PEPA**, Bénédicte **DEAU FISCHER**, Elizabeth **DE KORT**, Erik **DE CABO**, François **DANION**, Etienne **DAGUINDAU**, Tania **CUSHION**, Louise **CREMER**, Marianna **CRISCUOLO**, Gregorio **CORDINI**, Antonella **CINGOLANI**, Fabio **CICERI**, Fazle Rabbi **CHOWDHURY**, Ekaterina **CHELYSHEVA**, Adrien **CHAUCHET**, Louis Yi Ann **CHAI**, M. Mansour **CEESAY**, Elena **BUSCH**, Mathias **BREHON**, Davimar M.M. **BORDUCCHI**, Stephen **BOOTH**, Serge **BOLOGNA**, Caroline **BERG VENEMYR**, Rebeca **BAILÉN-ALMOROX**, Anastasia **ANTONIADOU**, Amalia N. **ANASTASOPOULOU**, Fevzi **ALTUNTAŞ**
